# Allosteric Tuning of Caspase‐7: A Fragment‐Based Drug Discovery Approach

**DOI:** 10.1002/anie.201706959

**Published:** 2017-10-09

**Authors:** Nicholas R. Vance, Lokesh Gakhar, M. Ashley Spies

**Affiliations:** ^1^ Division of Medicinal and Natural Products Chemistry College of Pharmacy University of Iowa 115 S Grand Ave Iowa City IA 52242 USA; ^2^ Department of Biochemistry College of Medicine University of Iowa 51 Newton Road Iowa City IA 52242 USA; ^3^ Protein Crystallography Facility Roy J. and Lucille A. Carver College of Medicine University of Iowa 51 Newton Road Iowa City IA 52242 USA

**Keywords:** allostery, apoptosis, caspases, drug discovery, enzyme catalysis

## Abstract

The caspase family of cysteine proteases are highly sought‐after drug targets owing to their essential roles in apoptosis, proliferation, and inflammation pathways. High‐throughput screening efforts to discover inhibitors have gained little traction. Fragment‐based screening has emerged as a powerful approach for the discovery of innovative drug leads. This method has become a central facet of drug discovery campaigns in the pharmaceutical industry and academia. A fragment‐based drug discovery campaign against human caspase‐7 resulted in the discovery of a novel series of allosteric inhibitors. An X‐ray crystal structure of caspase‐7 bound to a fragment hit and a thorough kinetic characterization of a zymogenic form of the enzyme were used to investigate the allosteric mechanism of inhibition. This work further advances our understanding of the mechanisms of allosteric control of this class of pharmaceutically relevant enzymes, and provides a new path forward for drug discovery efforts.

The caspase family of enzymes are responsible for controlling the final steps of inflammation and various cell death pathways. This highly regulated family of endoproteases employ a catalytic cysteine/histidine dyad to hydrolyze peptides with high specificity for aspartate at the P1 position.^1^ Initiator caspases (−8, −9) are activated in response to apoptotic stimuli to further activate executioner caspases (−3, −6, −7) by proteolytically cleaving the aspartate‐containing inter‐linker subunit (see the Supporting Information, Figure S1). The activity of these dimeric caspases is tightly regulated through numerous avenues: expression as a zymogen, post‐translational modifications, and regulatory proteins.^2^ Once activated, they carry out the final steps of apoptosis by judiciously cleaving hundreds of substrates to dismantle the cell in a controlled fashion.

Dysregulation of caspase activity has been implicated in numerous neurodegenerative, inflammatory diseases and cancers.^3^ Most efforts directed towards the development of caspase therapeutics have focused on targeting the active site. This is problematic because of the caspase's preference for negatively charged substrates, which inevitably leads to small‐molecule inhibitors with poor physicochemical properties. Wells and co‐workers discovered an allosteric site at the dimer interface of the caspases by employing a fragment tethering approach.^4^ This method relies on the formation of a disulfide bond between the receptor cysteines and small molecules containing thiols. These classes of compounds serve as useful probes, but do not represent promising fragment leads.

High‐throughput screening methods have yet to yield drug leads that are capable of allosterically inhibiting caspases. It is possible that current screening libraries do not contain an appropriate complementary chemical space. Over the last twenty years, fragment‐based drug discovery (FBDD) has emerged as a powerful, complementary method for the generation of new chemical hits.^5^ Typical high‐throughput screening methods attempt to identify potent chemical leads (<10 μm) by screening upwards of millions of rule‐of‐five compliant small molecules (<500 Da).^6^ By screening small libraries of hundreds to thousands of fragment molecules (<300 Da), it is possible to sample far greater chemical diversity.^7^ While fragments identified by FBDD have low affinity (μm–mm), they possess higher ligand efficiency and represent strong foundations for drug discovery efforts.

The current study employed a FBDD approach to identify a series of fragments capable of allosterically inhibiting caspase‐7 (C7). To gain insight into the nature of this long‐range allosteric inhibition, we measured the p*K*_a_ values of essential catalytic residues and compared the solvent isotope effects between C7 and the constitutive zymogen, procaspase‐7 D198A, D206A (P7‐D_2_A). These data suggest that the source of the allosteric inhibition is the movement of the catalytic dyad at positions C186 and H144 into non‐optimal geometries, which results in a slow acylation step relative to the mature caspase C7. Overall, these structural and biochemical results clearly reveal a way forward for effectively inhibiting executioner caspases with reversible drug‐like lead compounds that do not directly compete with substrate.

Fragment screening against caspase‐7 resulted in the identification of two novel, drug‐like, non‐competitive inhibitors. X‐ray crystallography confirmed that these compounds bind at the dimer interface of caspase‐7 more than 17 Å from the active site. Fragments that are chemically similar to these initial hits were purchased and assayed, and found to have increased ligand efficiency and potency. Our crystal structure of the C7–inhibitor complex revealed a number of distortions, including the position of the catalytic C186 and the substrate‐binding loops. This study provides a path forward for the development of caspase inhibitors while advancing our understanding of the allosteric mechanism of control in this important class of enzymes.

A 1000 compound fragment library from Maybridge was screened against caspase‐7 by differential scanning fluorimetry (DSF).^8^ This primary screen resulted in several validated biophysical hits exhibiting a non‐competitive mechanism of inhibition. The effect on enzymatic activity was assessed by hydrolysis of the preferred peptide fluorogenic substrate, Ac‐DEVD‐7‐AFC. Compounds **1** and **2** were found to have IC_50_ values of 3.98±1.05 mm and 8.52±1.20 mm, respectively (Figure 1 A). Steady‐state kinetic analysis of C7 with **1** showed a concentration‐dependent lowering of the maximal velocity (*V*_max_) while the Michaelis constant (*K*_m_) remained unchanged (Figure 1 B). These results are consistent with a non‐competitive mechanism of inhibition with inhibition constants (*K*_i_) of 5.5±0.40 mm and 4.3±0.20 mm for **1** and **2**, respectively (Table 1). In contrast to their thiol‐ or transition‐metal‐containing predecessors, these hits are drug‐like fragments that inhibit C7 in a reversible and non‐competitive manner.^4^, ^9^

**Figure 1:**
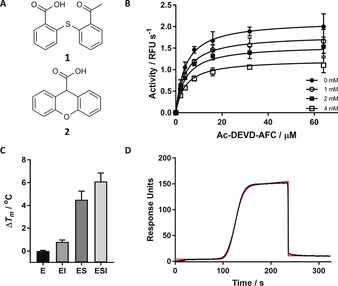
A) Chemical structures of **1** and **2**, identified from fragment screening. B) Michaelis–Menten kinetics. In the presence of increasing concentrations of **1**, there is an apparent lowering of *k*_cat_, while *K*_m_ is relatively constant. This is consistent with a non‐competitive mechanism of inhibition. C) DSF of C7 in the presence of DMSO (E), **1** (EI), substrate Ac‐VAD‐FMK (ES), and both **1** and Z‐VAD‐FMK (ESI) shown in the bar graph on the right in triplicate. D) Binding of **1** to C7 as measured by SPR. OneStep injection was fit with a simple 1:1 binding model shown in red, raw data are shown in black. Further details on the OneStep injection and analysis can be found in the Supporting Information.

**Table 1:** Summary of data from the Ac‐DEVD‐AFC cleavage assay for inhibitors containing a thiophenol core. IC_50_ (μm) and *K*_i_ (μm) data were obtained in Ac‐DEVD‐AFC cleavage assays. The ligand efficiency (LE) represents the binding affinity per non‐hydrogen atom (HA) in units of kcal mol^−1^/HA.

Compound		IC_50_ [μm]	*K*_i_ [μm]	LE^[a]^ [kcal mol^−1^ HA^−1^]
**1**		3980±1.05	5470±398	0.18
**2**		8520±1.20	4320±194	0.17
**3**	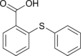	2190±1.06	1970±48.0	0.23
**4**	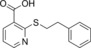	3500±1.05	2270±102	0.19
**5**	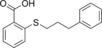	930±1.08	913±30.4	0.22
**6**	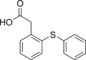	637±1.04	667±31.9	0.26

[a] LE=Δ*G*/HA=−1.4(pIC_50_)/HA; where the binding affinity was measured in terms of the IC_50_ value.^7^

A rudimentary DSF experiment was used to show that **1** is indeed capable of binding the free enzyme or the enzyme–substrate complex, as suggested by the non‐competitive model of inhibition (Figure 1 C). In the presence of either substrate or **1**, the melting temperature (*T*_m_) increased markedly relative to the control. However, the ESI complex appeared to show an additive effect of simultaneous binding. This indicates that the allosteric inhibitor is not competing (even indirectly) for binding with substrate. Interestingly, several disulfide‐forming allosteric caspase inhibitors were shown to have a competitive mechanism of inhibition as the binding of substrate or inhibitor was mutually exclusive.^4^

Surface plasmon resonance (SPR) was used to confirm the binding of the lead fragment hits to C7. The protein was fixed to the surface of a HisCap biosensor via the 6x‐His tag, as detailed in the Supporting Information. Figure 1 D shows the binding of **1** to C7 during an SPR OneStep injection (see the Supporting Information for details). Dissociation constants of 8.00±3.0 mm and 4.09±0.3 mm measured for **1** and **2**, respectively, agreed well with the inhibition data (Table S1). The SPR data were fit with a 1:1 binding stoichiometry, and the magnitude of the signal measured was consistent with this stoichiometry.

Fragments similar to **1** were purchased from commercial vendors to try and identify more potent inhibitors. The majority of this series of fragments yielded improved affinity and a modest increase in ligand efficiency (Table 1). Of this second series, **6** was found to have an IC_50_ value of 637±1.04 μm with a 44 % increase in ligand efficiency (LE). Fragment **6** is now approaching the ideal LE of 0.3, in contrast to the hits **1** and **2** from the primary screen.^7^ This fragment is the most potent non‐competitive inhibitor discovered thus far and represents an attractive starting point for further structure‐based optimization.

The X‐ray crystal structure of caspase‐7 soaked with **1** was determined at a resolution of 2.8 Å (Table S2). During soaking experiments with **1**, electron density appeared in the known allosteric site at the dimer interface of C7 near K160 (Figure 2 A). The carboxyl group in **1** forms an ionic interaction with the neighboring R167, and a hydrogen bond with T163 (Figure S2). The binding of **1** occurred more than 5 Å away from C290, the residue utilized for the tethering of thiol‐containing fragments, further supporting the reversibility of the binding of these fragments.^4^ The appearance of density in the allosteric site occurred concurrently with the movement of the catalytic cysteine C186 into the P1 substrate‐binding pocket and the disappearance of the active‐site loop density for loops L1, L2, L3, L4, and L2′ (Figure 2 B–D). These results are consistent with what has been seen with other allosterically inhibited C7 structures.^4^, ^9^

**Figure 2:**
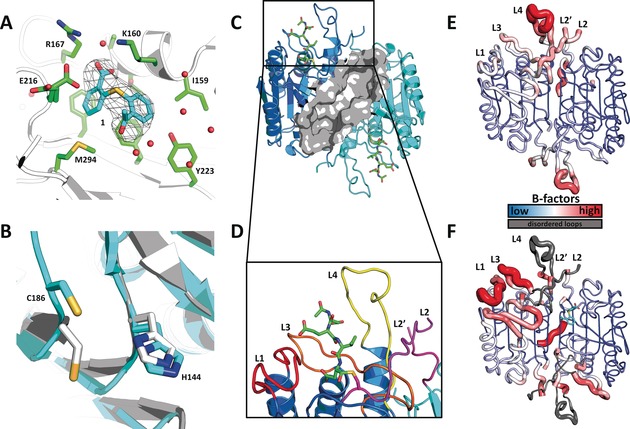
A) Crystal structure of C7 soaked with **1** (PDB ID: 5V6U), polder *F*_o_−*F*_c_ omit map contoured at 5.0 σ r.m.s. (root mean square) and carved 5 Å around **1** (cyan sticks) at the dimer interface; neighboring residues (<8 Å) are shown as green sticks and water molecules as red spheres. B) Alignment of catalytic C186/H144, shown in sticks, for mature C7 (4FDL, cyan) and C7 bound to **1** (5V6U, light gray/white). C) Overview of the C7 dimer bound to substrate (PDB ID: 1F1J). Colors highlight monomer A (blue), monomer B (cyan), peptide substrate Ac‐DEVD‐CHO (green sticks), and the allosteric site (gray surface). D) Close‐up view of the active‐site loop bundle. The Ac‐DEVD‐CHO peptide substrate is shown as green sticks, loops are colored red (L1), orange (L3), yellow (L4), and magenta (L2/L2′), and L2′ is donated from the opposite monomer. Cartoon representation of E) active C7 and F) C7 bound to **1** (cyan sticks; 5V6U), colored according to their normalized B‐factors from low to high (blue to red). Dark gray loops for 5V6U represent disordered regions not modelled into the crystal structure and are overlaid from 4FDL for reference.

Comparison of normalized B‐factors from C7 and the allosterically inhibited form highlights the mobility of the active‐site loops upon inhibitor binding (Figure 2 E, F). Binding of fragment **1** appeared to move E216 towards Y229, displacing it and consequently the L2 loop (Figure S3). The partial occupancy of 0.71 for **1** results in alternate rotamer conformations of E216 (Figure 2 A). It has previously been demonstrated by Hardy and Witkowski that the L2/L2′ loop bundle is critical for C7 activity.^10^ Mutations in the L2 loop have been shown to reduce the activity through *k*_cat_ and/or *K*_m_ effects.^10^, ^11^

Electron density was obtained for only one ligand in each dimer, near one monomer. The proposed stoichiometry for binding would be 1:1 per monomer based on the fact that there is no known cooperativity between executioner caspase dimers. The protomers of the dimer have significantly different crystal packing contacts in the unit cell (Figure S4). A comparison of the B‐factors between chains A and B for C7 bound to **1** (5V6U) and C7 (4FDL) shows that the small molecule binds to the more stable of the two chains (Figure S5/6).

It has been hypothesized that compounds binding to the allosteric site are capable of shifting the conformation of the caspase‐7 enzyme towards a zymogen‐like state.^12^, ^13^, ^14^ There is conflicting literature regarding the activity of executioner caspase zymogens. Wells and co‐workers found that the zymogen *K*_m_ value is significantly higher, while the *k*_cat_ is strongly reduced.^15^ Other studies found that the procaspase‐3 zymogen had an unchanged *K*_m_, with a significantly reduced *k*_cat_ value.^16^, ^17^, ^18^ To further investigate the mechanisms of allosteric inhibition in C7, we expressed the mutant P7‐D_2_A, a constitutive zymogen. The *k*_cat_/*K*_m_ vs. pH profile did not conform to a bell shape, rather there were two distinct enzyme forms and three apparent ionizations over the pH range tested, as seen with caspase‐3 (Figure S7).^17^ The main differences in activity for C7 and P7‐D_2_A resulted from differences in *k*_cat_, rather than *K_m_* (Figures S8 and S9). The *k*_cat_/*K*_m_ profiles for C7 and P7‐D_2_A had three ionization states of 5.6, 9.0, and 11.76 and 5.3, 7.3, and 10.4, respectively.

The prototypical cysteine protease, papain, is the most thoroughly understood and was used here to rationalize the kinetic data. The catalytic cysteine/histidine dyad of papain was assigned p*K*_a_ values of 4.0 and 8.5, respectively.^19^ As papain is a lysosomal protease with an optimal pH value of around 4–5, the enzyme rests as a thiolate–imidazolium ion pair. If caspase acted through a similar mechanism, then the lowest ionization states of 5.6 and 5.3 could be assigned to C186 for C7 and P7‐D_2_A, respectively. The intermediate p*K*_a_ values of 9.0 for C7 and 7.3 for P7‐D_2_A likely correspond to H144. Indeed, in this scenario, the zymogen would possess a significant portion of the catalytic histidine in the uncharged form under physiological conditions, which would be expected to significantly slow down the acylation step. Finally, the third ionization state of 11.8 and 10.4 for C7 and P7‐D_2_A, respectively, was unexpected, but could be explained by a critical R187 neighboring the catalytic C186, or K212 in the L2/L2′ loop bundle.^10^, ^11^

Finally, solvent isotope effects (SIEs) were used to further dissect the allosteric caspase regulation. As expected, the mature caspase‐7 has a large ^D^ *k*_cat_ value of 4.11, and an inverse isotope effect on ^D^(*k*_cat_/*K*_m_) of 0.92 (Table S3). This indicates that the rate‐limiting step of the catalysis is indeed the hydrolysis of the acylated enzyme. Enrichment of the thiolate form of cysteine proteases in D_2_O is thought to cause the inverse isotope effect on ^D^(*k*_cat_/*K*_m_).^20^ The SIE on P7‐D_2_A was smaller, with ^D^ *k*_cat_ and ^D^(*k*_cat_/*K*_m_) values of 2.62 and 0.45, respectively (Table S3). Assuming that the rate‐limiting step for catalysis with the wild‐type enzyme is deacylation, as is the case for most cysteine proteases, it appears that the observed SIE is reduced in the zymogen as there is a slower chemical or physical step (which is isotopically insensitive). This lower expression of the SIE shows that the energetic barriers are altered in the zymogen. This is consistent with the hypothesis that the zymogenic form of the enzyme has a much slower acylation step as the Cys–His catalytic dyad is 1) not fully present as the ion pair and 2) may be in a poor geometry for acylation. This is also consistent with the observed effect of no change in the *K*_m_ value of the substrate, but rather a purely *k*_cat_ effect.

The compounds discovered in this study represent the first inhibitory, drug‐like fragments to target an executioner caspase. Further investigations through chemical similarity metrics yielded more potent compounds with yet even higher ligand efficiency. The majority of screening efforts against the caspase family of hydrolases has focused on HTS methods. These efforts have motivated the screening of hundreds of thousands of small molecules but have yet to yield tractable lead scaffolds for the development of caspase‐targeted therapies. This further highlights the utility of FBDD for generating momentum in a drug discovery campaign by leveraging superior chemical diversity.

The distortion of the substrate‐binding loops, coupled with the movement of the Cys/His dyad, led us to hypothesize that the allosteric mechanism of inhibition could be mediated through changes in the p*K*_a_ values of the essential residues for catalysis, yielding new rate‐limiting steps for catalysis. By using the surrogate P7‐D_2_A, we have demonstrated that indeed the p*K*_a_ values of the catalytic residues have shifted, and SIE experiments indicated that a new isotopically insensitive rate‐limiting step had been introduced. By studying the allosteric mechanisms of control for this class of hydrolases, this knowledge can be used to guide the development of this new class of caspase inhibitors.

## Conflict of interest

The authors declare no conflict of interest.

## Supporting information

As a service to our authors and readers, this journal provides supporting information supplied by the authors. Such materials are peer reviewed and may be re‐organized for online delivery, but are not copy‐edited or typeset. Technical support issues arising from supporting information (other than missing files) should be addressed to the authors.

Supplementary
