# Supplementary material for: Allosteric Tuning of Caspase‐7: A Fragment‐Based Drug Discovery Approach
Source: Angew Chem Int Ed Engl. 2017 Oct 9;56(46):14443–7. doi: 10.1002/anie.201706959 (PMC5698726; doi:10.1002/anie.201706959)

Angewandte Chemie
Eine Zeitschrift der Gesellschaft Deutscher Chemiker

Supporting Information

Allosteric Tuning of Caspase-7: A Fragment-Based Drug Discovery Approach

*Nicholas R. Vance, Lokesh Gakhar, and M. Ashley Spies**

anie_201706959_sm_miscellaneous_information.pdf

Author Contributions

N.V. Conceptualization: Equal; Data curation: Lead; Formal analysis: Lead; Funding acquisition: Equal; Investigation: Equal; Methodology: Lead; Software: Lead; Validation: Lead; Visualization: Lead; Writing—original draft: Lead; Writing – review & editing: Lead
M.S. Conceptualization: Equal; Data curation: Supporting; Formal analysis: Equal; Funding acquisition: Lead; Investigation: Lead; Methodology: Equal; Project administration: Lead; Resources: Lead; Supervision: Lead; Writing—original draft: Equal; Writing—review & editing: Equal
L.G. Data curation: Supporting; Formal analysis: Supporting; Software: Supporting; Supervision: Supporting; Validation: Supporting; Writing—review & editing: Supporting.

Table of Contents

Supplementary methods……………………………………………………………………..02-08
Supplementary figures…………………………………………………………………….09-17
Supplementary tables……………………………………………………………………...18-21
Compound characterization……………………………………………………………22-34

Materials and Methods

Chemicals and reagents

The fragment library was purchased from Maybridge, and the structurally related, follow up compounds were purchased from Maybridge. The 1000 compound core diversity fragment set from Maybridge was used for screening and guaranteed ≥95% purity and NMR for each compound. The caspase substrate Ac-DEVD-AFC was purchased from Enzo Life Sciences, Inc.

Plasmids, recombinant protein expression and purification

Full-length, human procaspase-7 with a C-terminal his-tag in a pET23b vector (Novagen) was received as a gift from Guy Salvesen (Addgene plasmid #11825).[1] A constitutive procaspase-7 mutant (D198A, D206A) was created through site-directed mutagenesis, and both the wild type and mutant caspase sequences were confirmed through sanger sequencing at the Iowa Institute of Human Genetics Genomics Division. Both the procaspase-7 wild type and double mutant were expressed from *E. coli* BL21 (DE3) pLysS (Stratagene) in pET23-b vectors with a C-terminal 6x-His-tag as previously described.[2] Cells were grown at 37 ºC and 175 rpm to a density of OD_{600} = 0.8-1.0 in 2xYT media with 200 µg mL^{-1} ampicillin (pET23b vector) and 50 µg mL^{-1} chloramphenicol (pLysS), at which time protein expression was induced with the addition of 0.2 mM IPTG. Procaspase-7 wild type was expressed at 37 ºC for 16-18 hours overnight to allow for auto-activation to the fully mature, caspase-7. Procaspase-7 D_2A was expressed for 2 hours at 30 ºC to prevent fortuitous cleavage of the intersubunit linker by *E. coli* proteases. After expression, cells were pelleted by centrifugation at 5,000 x g and 4 ºC for 20 min. All subsequent purification steps were performed at 4 ºC to prevent sample degradation. Cell pellets were resuspended in buffer A (100 mM Tris, 100 mM NaCl, 10 mM imidazole, pH = 8, 1 mM TCEP). PMSF was added to a final concentration of 1 mM to prevent serine protease degradation of the sample, and DNaseI (EMD Millipore) was added to reduce sample viscosity. An emulsiflex microfluidizer was used to lyse the cells, the cell suspension was passed through the instrument three times. The lysate was clarified by centrifugation at 30,000 x g for 60 min and passed through a 0.22 µm filter before being loaded onto a 1 mL HiTrap IMAC HP (GE Healthcare) nickel resin equilibrated with buffer A at approximately 1 mL min^{-1}. The column was washed with 10 column volumes (CV) of buffer A before a linear gradient of 30 CV from 100% buffer A to 100% buffer B (100 mM Tris, 100 mM NaCl, 250 mM imidazole, pH = 8, 1 mM TCEP) and then held at 100% B for 10 CV. The fractions of interest identified by 15% SDS-PAGE were concentrated to < 2.0 mL using a 10 kDa cutoff Amicon centrifugal concentrator (EMD Millipore) and injected onto a HiLoad Superdex 200 PG (16/600) gel filtration column (GE Healthcare). The S200 gel filtration column was equilibrated and the protein was eluted with the same protein storage buffer (20 mM Tris, 50 mM NaCl, pH = 8.0, 5 mM DTT) at 1.0 mL min^{-1}. A gel filtration standard of proteins ranging from 1,350 to 670,000 Da (Bio-Rad) was run to create a calibration curve of molecular weight versus elution volume. As expected, the C7 proteins eluted as an approximately 60 kDa dimer. Again, fractions of interest as assessed by 15% SDS-PAGE gels were pooled and concentrated to 1 mg mL^{-1} and stored at -20ºC. Protein concentration was assessed by absorbance at 280 nm using the Edelhoch relationship and a molar extinction coefficient of ε_{280} = 24,510 M^{-1} cm^{-1}.[3-4]

Enzyme activity assays

Caspase-7 activity was assessed by the liberation of the aminofluorocoumarin from the fluorogenic substrate Ac-DEVD-AFC (Enzo Life Sciences) in a standard caspase assay buffer consisting of 20 mM PIPES, pH = 7.5, 100 mM NaCl, 1 mM EDTA, 10 mM B-mercaptoethanol, 0.1%

(w/v) CHAPS, 10% (w/v) sucrose.[5] Measurements were carried out in black 96-well plates (Costar) in a total volume of 100 μL per well. Caspase-7 (10 nM) was incubated in caspase assay buffer with either DMSO (< 4%) or compound (200 mM DMSO stock) for 15 min, then the reaction was initiated by the addition of substrate. The reaction was monitored by fluorescence emission (λ_{ex} = 400 nm, λ_{em} = 505 nm) at 25°C for 5 min. Absorbance spectra (200-800 nm) were collected at the highest compound concentration to assess potential inner filter effect, and DMSO%(v/v) was normalized.

The concentration of caspase active sites was determined by titration of an irreversible inhibitor Z-VAD-FMK (Enzo Life Sciences).[5] The protein was incubated at a high concentration with a small amount of DMSO solvated Z-VAD-FMK to facilitate the active site labeling, prior to diluting into the standard caspase assay buffer lacking reducing agent. The active sites were titrated by plotting the residual enzymatic activity against the concentration of Z-VAD-FMK. Linear regression was performed to determine the x-intercept, which was used as the concentration of active sites in the preparation. Graphpad Prism (7.01) was used to fit dose-response curves, michaelis-menten kinetics, and non-competitive inhibition.

Differential Scanning Fluorimetry

DSF experiments were performed in 96-well polypropylene PCR plates (Bio-Rad MLL9601) on a Bio-Rad CFX96 Touch Real-Time PCR instrument. Assay components included C7 (10 μM), Sypro Orange dye (10x), and compound from DMSO stocks (< 4%), with a final volume of 25 μL.[6] Experiments were carried out with a temperature gradient of 1 °C min^{-1} from 25 °C to 95 °C. Melting data was processed with the java applet DMAN.[7]

Fragments that were capable of increasing the melting temperature (T_m) more than 0.50 ºC relative to DMSO control were considered hits. The T_m of a protein melting curve was defined as the relative maximum of the first derivative of the melting curve. Compounds that exhibited unusually high background fluorescence or deviated from two-state unfolding kinetics were triaged.

Surface Plasmon Resonance

Surface plasmon resonance (SPR) measurements were performed on a SensiQ (Oklahoma City, OK, USA) Pioneer AE instrument. C-terminal, hexa-histidine tagged caspase-7 wild type was immobilized to a nickel-nitriloacetic acid coated 3D HisCap biosensor (SensiQ, Oklahoma City, OK, USA) New biosensor chips were first "seasoned" in preparation for protein binding. Seasoning consists of first injecting 50 mM NaOH at 40 μL min^{-1} for 30 sec, then 0.1% (w/v) SDS at 40 μL min^{-1} for 30 sec, then 10 mM HCl for 30 sec, all with fast injection types. This cycle of NaOH, SDS, and HCl injections are repeated to help "fluff" the matrix in preparation for immobilization. Protein immobilization and kinetics of ligand binding were assessed in an SPR buffer consisting of 50 mM Hepes, pH = 7.5, 100 mM NaCl, 1 mM TCEP, 0.01% Triton-X100, 1% PEG-3350, and 4%(v/v) DMSO.[8] All buffers and solutions were filter sterilized with 0.22 μm buffer filters before using on the SPR instrument. After seasoning, the lines were primed three times with SPR buffer.

The HisCap chip was prepped for protein immobilization by washing with 200 μL of 100 mM EDTA (pH=8.0), at 20 μL min^{-1}, then activated with 100 μM nickel (II) chloride for 2 min at 40 μL min^{-1}, both with fast injection types. The channel was then cleaned with 200 μL injection of 700 mM imidazole at 20 μL min^{-1}, then a 200 μL injection of an approximately 50 μg mL^{-1} solution of caspase-7 was injected onto the channel at a flow rate of 10 μL min^{-1} fast injection types. Protein is injected across the nickel activated channel until it reaches the desired response units (RU) of immobilized protein, approximately 8,000 – 12,000 RU. This density of protein on the biosensor surface is necessary for measuring binding interactions of fragment size small molecules (<300 Da) The remaining protein is washed out with SPR buffer and buffer is continuously pumped until the baseline

is stabilized with a drift of no more than 3 RU min^{-1}. Typically we would immobilize between 8-10,000 RU of protein onto the surface of the chip.

OneStep™ injections were used to determine the dissociation constant (K_d), as well as the microscopic rate constants for association (k_a) and dissociation (k_d) of the protein ligand interactions. Consistent with SPR terminology, the protein immobilized onto the surface of the chip is referred to as the "ligand" and the molecule flowed into the channel is referred to as the "analyte". The SensiQ Pioneer AE is capable of performing OneStep™ injections where analyte is diluted in-line with buffer to create a concentration gradient injection that allows full characterization of binding kinetics in a single injection. Calibrations of the concentration gradient dilution were performed with 20% (w/v) sucrose injections.

The SensiQ OneStep injection is essentially a concentration gradient injection of compound that allows a full characterization of the microscopic rate constants k_a and k_d as well as the dissociation constant, K_d. The binding observed was consistent with 1:1 stoichiometry for the binding of the fragments to each monomer. The measured response units for a binding interaction is dictated by the equation $RU_{max} = RU_{ligand} \times (MW_{analyte}/MW_{ligand}) \times V_{ligand}$; where the ligand is the protein immobilized on the surface and the analyte is the fragment, and RU_{max} is the expected response units for a ligand saturated with analyte, RU_{ligand} is the response units of ligand immobilized on the surface, $MW_{analyte}$ is the molecular weight of the analyte, MW_{ligand} is the molecular weight of the ligand, and V_{ligand} is the stoichiometry of the binding interaction. Based on the amount of protein immobilized on the surface of the chip, the RU_{max} measured for the inhibitors is consistent with a 1:1 stoichiometry. Also, there is no known cooperativity between the active sites of the dimeric caspase family of enzymes, so inhibition would require at least 1:1 binding. All sensorgrams were analyzed with Qdat software.

pH rate profiles

The pH dependence of C7 and P7-D_2A was measured by the hydrolysis of Ac-DEVD-AFC at 25 ºC in buffers ranging from pH of 5.5-10.5. The following buffers were used for each pH range: 20 mM MES (5.5-6.5), 20 mM HEPES (6.2-7.3), 20 mM PIPES (6.9-8.1), 20 mM Bicine (7.8-9.0), 20 mM CHES (8.8-10.0), and 20 mM CAPS (9.7-11.1), and the optimized caspase assay buffer conditions included 100 mM NaCl, 1 mM EDTA, 0.1% (w/v) CHAPS, and 10% (w/v) sucrose, and fresh 5 mM β-mercaptoethanol added before assays.[4] Buffer pH was adjusted with NaOH or HCl. GraphPad Prism (7.01) was used to fit the michealis-menten kinetics parameters, k_{cat} and K_m, by non-linear regression. The pH range was kept above 5 because caspase-3 is monomeric below that pH, and since caspase-3 and -7 have a sequence identity of 56% and a sequence similarity of 73%, we sought to ensure that the protein was fully dimeric over the pH range tested.[9-10] Points above pH=10.5 were excluded due to the increasingly high concentrations of substrate needed to accurately measure k_{cat}.

Equations 1-3, shown below, were used to fit the pH dependence of k_{cat}/K_m for C7 and P7-D_2A; where $(k_{cat}/K_m)_{app}$ is the measured kinetic parameter, $(k_{cat}/K_m)_o$ and $(k_{cat}/K_m)'_o$ are the pH-independent values of k_{cat}/K_m, [H] is the proton concentration, and K_{e1}, K_{e2}, K_{e3} are the proton dissociation constants.[11-12] The equations for pH dependence of k_{cat}/K_m were fit by the non-linear least-squares method in GraphPad Prism 7 (version 7.01). Equations 1 and 2 represent bell-shaped curves for enzymes dependent on two or three ionizable groups, respectively. After initial attempts to fit these pH-rate profiles with equations 1 and 2, it became apparent there were two distinct maxima and the data did not conform to a bell shaped curve. The data conformed to a model described by equation 3, where there are two pH-independent values of k_{cat}/K_m represented by two forms of the enzyme with different ionization states, with three total ionizable groups. This latter equation seems to be a reasonable model owing to the fact that the L2/L2' loops of caspase-7 contain ionizable groups known to allosterically regulate this enzymes' activity.

$$[Eq.(1)]\ (k_{cat}/K_m)_{app} = \frac{(k_{cat}/K_m)_o}{1 + \frac{[H]}{K_{e1}} + \frac{K_{e2}}{[H]}}$$

$$[Eq.(2)]\ (k_{cat}/K_m)_{app} = \frac{(k_{cat}/K_m)_o}{1 + \frac{[H]}{K_{e1}} + \left(\frac{K_{e2}}{[H]}\right)\left(1 + \frac{K_{e3}}{[H]}\right)}$$

$$[Eq.(3)]\ (k_{cat}/K_m)_{app} = \frac{(k_{cat}/K_m)_o}{1 + \frac{K_{e2}}{[H]} + \frac{[H]}{K_{e3}} + \frac{[H]^2}{K_{e3}K_{e1}}} + \frac{(k_{cat}/K_m)'_o}{1 + \frac{[H]}{K_{e1}} + \frac{K_{e3}}{[H]} + \frac{K_{e2}K_{e3}}{[H]^2}}$$

Crystallization, X-ray data collection and structure determination

Crystals of unliganded caspase-7 were grown using the hanging-drop vapor diffusion method at room temperature, approximately 23 ºC. Crystallization conditions were focused around 0.1 M sodium citrate, pH=5.0-5.8, with a sodium formate precipitant concentration of 2.1 M.[13] The protein solution was concentrated to 6-9 g L^{-1} as assessed by absorbance at 280 nm by the edelhoch relationship, vide supra. After protein concentration, the solution was centrifuged at 13,300 x g for 5 min at 4 ºC to pellet any insoluble material. Hanging drops were 2 µL total volume with a 1:1 ratio of protein:reservoir solution. Crystals formed overnight and grew to maximum dimensions of 400 x 200 x 200 µm over the course of two weeks. Tolerance of caspase-7 crystal formation for DMSO solvent content only allowed for a maximum 3% (v/v). However, the presence of a small amount of DMSO did decrease the nucleation rate, allowing for larger crystal growth. Unfortunately, the compounds were not soluble enough to allow for co-crystallization. Soaking was employed with high concentrations of **1**, upwards of 50 mM, for 1 to 72 hours. Soaking conditions were 10% (v/v) of 200 mM inhibitor stock in DMSO diluted directly into the reservoir solutions. Crystals were looped directly from soaking conditions and rapidly plunged into liquid nitrogen.

Diffraction data were collected remotely at the Advanced Light Source in Berkeley, CA at the macromolecular crystallography beamline 4.2.2. Data collection parameters were as follows; 0.1 º oscillation, 0.2 sec exposure time, 360 mm detector distance, wavelength of 1.001374 Å and at a temperature of 100 K. Data were indexed and integrated using XDS (2016-01-11), and merged and scaled with SCALA (3.3.22).[14-15] Molecular replacement for caspase-7 crystals soaked with compound 1 was performed with PHASER (2.5.7) using both protein chains A and B from PDB code 1F1J as the starting model.[16-17] Matthews coefficients and estimated solvent contents for soaked crystals were 3.0 and 59.6, respectively. Refinement calculations were carried out with PHENIX (1.11.1_2575) and model building was done in COOT (0.8.9).[18-19] Ligands were placed using the LigandFit program in PHENIX once the protein structure was modeled, and water was the last thing added to the structure. Polder omit maps were generated with PHENIX using the default parameters.[20] Data collection and refinement statistics are listed in Table S1. All protein structure figures were generated using PYMOL.[21]

Solvent isotope effects

Deuterated buffer consisting of 20 mM PIPES, 100 mM NaCl, 1 mM EDTA, 10 mM B-mercaptoethanol, 0.1% (w/v) CHAPS, and 10% (w/v) sucrose was used to measure the steady state

kinetics of caspase proteins in D_2O. Equation 4 was used to adjust the pD of the buffer to match the pH, by accounting for isotope effects on the pH meter.[22] Equations 5 and 6 describe the solvent isotope effects on the catalytic turnover (k_{cat}) and michaelis constant (K_m) in water and deuterium oxide. The solvent isotope effect is simply the ratio of the light over heavy atom rate constants of the respective steady state kinetic parameters.

$$[Eq.(4)]\ pD = pH + 0.4$$

$$[Eq.(5)]\ {}^{D}k_{cat} = \frac{{}^{H_2O}k_{cat}}{{}^{D_2O}k_{cat}}$$

$$[Eq.(6)]\ {}^{D}\left(\frac{k_{cat}}{K_m}\right) = \frac{{}^{H_2O}\left({}^{k_{cat}}/_{K_m}\right)}{{}^{D_2O}\left({}^{k_{cat}}/_{K_m}\right)}$$

References

[1] Q. Zhou, S. Snipas, K. Orth, M. Muzio, V. M. Dixit, G. S. Salvesen, *J Biol Chem* **1997**, *272*, 7797-7800.

[2] D. W. Wolan, J. A. Zorn, D. C. Gray, J. A. Wells, *Science* **2009**, *326*, 853-858.

[3] H. Edelhoch, *Biochemistry* **1967**, *6*, 1948-1954.

[4] H. R. Stennicke, G. S. Salvesen, *J Biol Chem* **1997**, *272*, 25719-25723.

[5] H. Roschitzki-Voser, T. Schroeder, E. D. Lenherr, F. Frolich, A. Schweizer, M. Donepudi, R. Ganesan, P. R. Mittl, A. Baici, M. G. Grutter, *Protein Expr Purif* **2012**, *84*, 236-246.

[6] E. H. Mashalidis, P. Sledz, S. Lang, C. Abell, *Nat Protoc* **2013**, *8*, 2309-2324.

[7] C. K. Wang, S. K. Weeratunga, C. M. Pacheco, A. Hofmann, *Bioinformatics* **2012**, *28*, 439-440.

[8] J. Murray, A. M. Giannetti, M. Steffek, P. Gibbons, B. R. Hearn, F. Cohen, C. Tam, C. Pozniak, B. Bravo, J. Lewcock, P. Jaishankar, C. Q. Ly, X. Zhao, Y. Tang, P. Chugha, M. R. Arkin, J. Flygare, A. R. Renslo, *ChemMedChem* **2014**, *9*, 73-77, 72.

[9] K. Bose, A. C. Clark, *Protein Sci* **2005**, *14*, 24-36.

[10] J. G. Walsh, S. P. Cullen, C. Sheridan, A. U. Luthi, C. Gerner, S. J. Martin, *Proc Natl Acad Sci U S A* **2008**, *105*, 12815-12819.

[11] J. L. Schneck, J. P. Villa, P. McDevitt, M. S. McQueney, S. H. Thrall, T. D. Meek, *Biochemistry* **2008**, *47*, 8697-8710.

[12] Y. Muta, H. Oneda, K. Inouye, *Biochem J* **2005**, *386*, 263-270.

[13] J. Agniswamy, B. Fang, I. T. Weber, *Apoptosis* **2009**, *14*, 1135-1144.

[14] W. Kabsch, *Acta Crystallogr D Biol Crystallogr* **2010**, *66*, 133-144.

[15] P. Evans, *Acta Crystallogr D Biol Crystallogr* **2006**, *62*, 72-82.

[16] A. J. McCoy, R. W. Grosse-Kunstleve, P. D. Adams, M. D. Winn, L. C. Storoni, R. J. Read, *J Appl Crystallogr* **2007**, *40*, 658-674.

[17] Y. Wei, T. Fox, S. P. Chambers, J. Sintchak, J. T. Coll, J. M. Golec, L. Swenson, K. P. Wilson, P. S. Charifson, *Chem Biol* **2000**, *7*, 423-432.

[18] P. D. Adams, P. V. Afonine, G. Bunkoczi, V. B. Chen, I. W. Davis, N. Echols, J. J. Headd, L. W. Hung, G. J. Kapral, R. W. Grosse-Kunstleve, A. J. McCoy, N. W. Moriarty, R. Oeffner, R.

J. Read, D. C. Richardson, J. S. Richardson, T. C. Terwilliger, P. H. Zwart, *Acta Crystallogr D Biol Crystallogr* **2010**, *66*, 213-221.

[19] P. Emsley, B. Lohkamp, W. G. Scott, K. Cowtan, *Acta Crystallogr D Biol Crystallogr* **2010**, *66*, 486-501.

[20] D. Liebschner, P. V. Afonine, N. W. Moriarty, B. K. Poon, O. V. Sobolev, T. C. Terwilliger, P. D. Adams, *Acta Crystallogr D Struct Biol* **2017**, *73*, 148-157.

[21] Schrodinger, LLC, **2015**.

[22] K. B. Schowen, R. L. Schowen, *Methods Enzymol* **1982**, *87*, 551-606.

Supplementary figures

Figure S1: Maturation of procaspase-7 (PDB ID: 1K88) to caspase-7 (PDB ID: 4FDL), from left to right, occurs after the proteolysis of the inter-subunit linker (orange). After cleavage, the L2 loop (orange) from one monomer forms a loop bundle with the L2’ loop (orange) from the opposite monomer. The homodimeric structures are colored blue and cyan for monomers A and B, respectively.

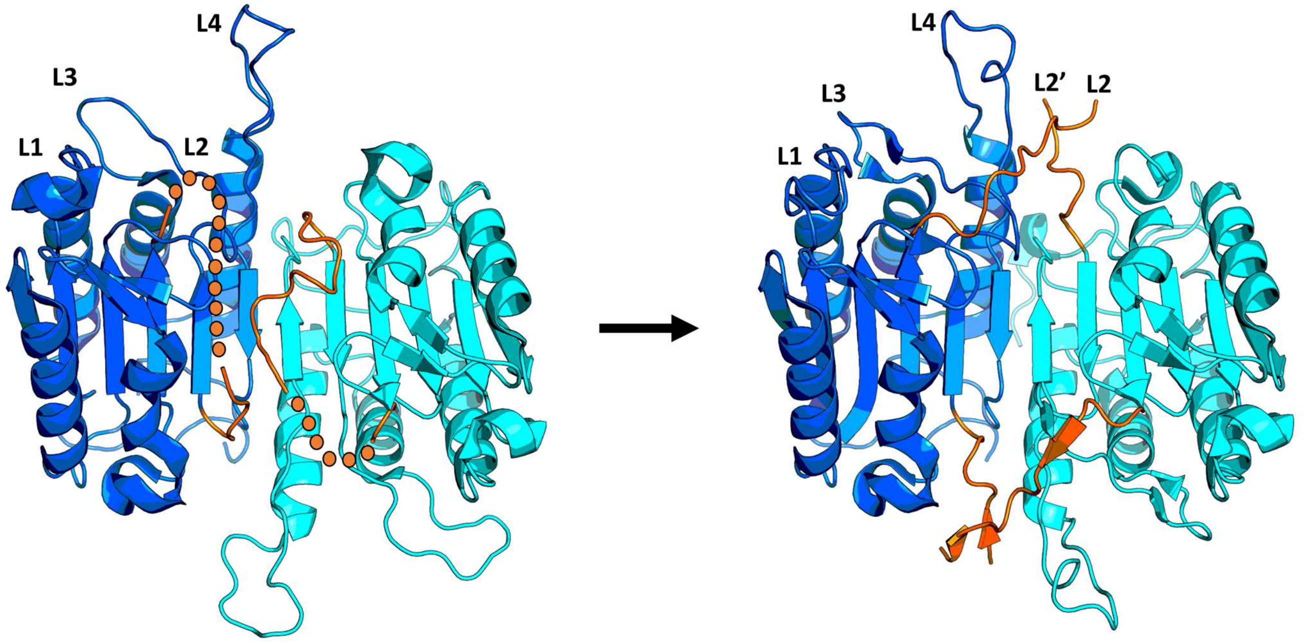

Figure S2: Fragment **1** binds near K160 in the allosteric site of C7 (PDB ID: 5V6U), with the carboxyl group in **1** forming a salt-bridge with the neighboring R167 and hydrogen-bonding interaction with T163 highlighted by yellow dashes with a distance measurement (Å).

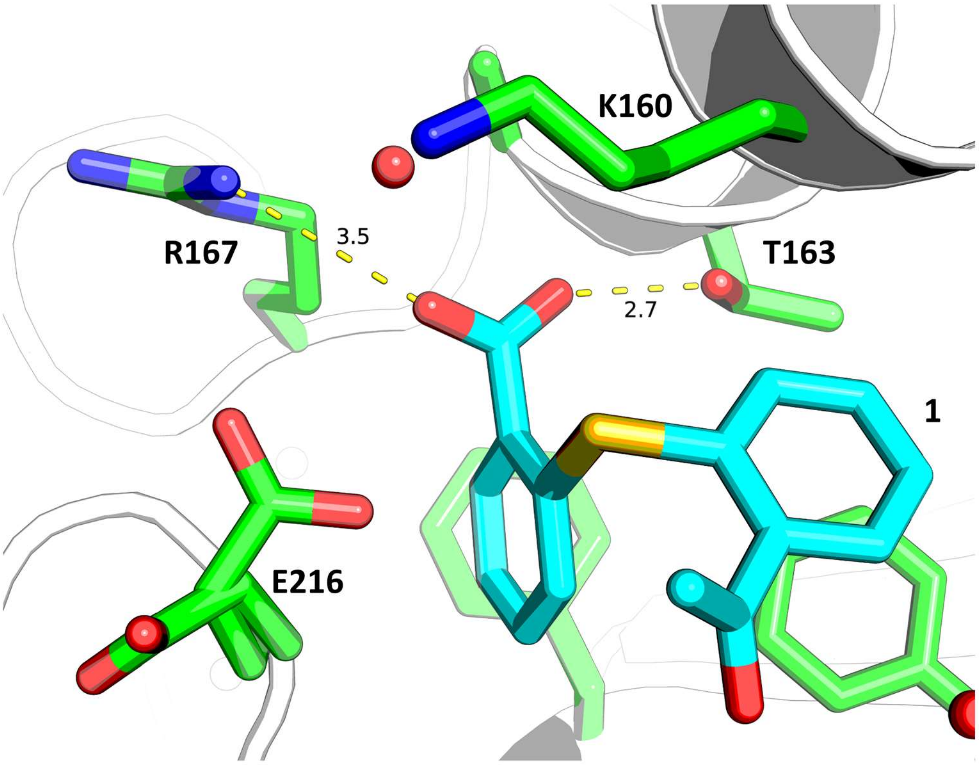

Figure S3: Binding of **1** (cyan sticks) to C7 displaces E216 towards the critical Y229 anchoring the L2 loop. The L2 loop bundle is critical for full caspase activity. Structure of C7 soaked with **1** (PDB ID: 5V6U, grey) is overlaid with the structure of mature caspase-7 (PDB ID: 4FDL, blue). Transparent Van der Waals spheres are shown to highlight clashing of residues.

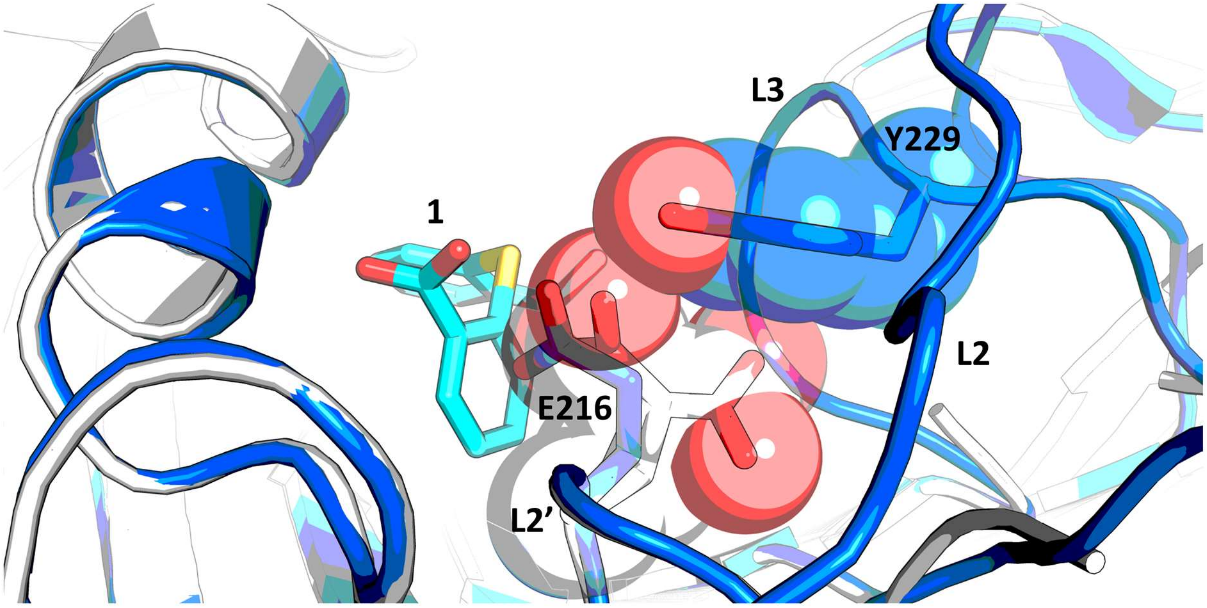

Figure S4: Structure of C7 bound to **1** (grey) with neighboring asymmetric units (yellow) from PDB ID: 5V6U. The small molecule **1** is shown in cyan bound to the dimer interface on chain B, the more ordered of the two monomers. Chain B is flanked by two crystallographic neighbors (Chain A' and Chain B'') which appear to be stabilizing the monomer. As soaking of the small molecule continues past 24-48 hours, the crystals begin to lose diffraction, presumably, as the second site becomes occupied.

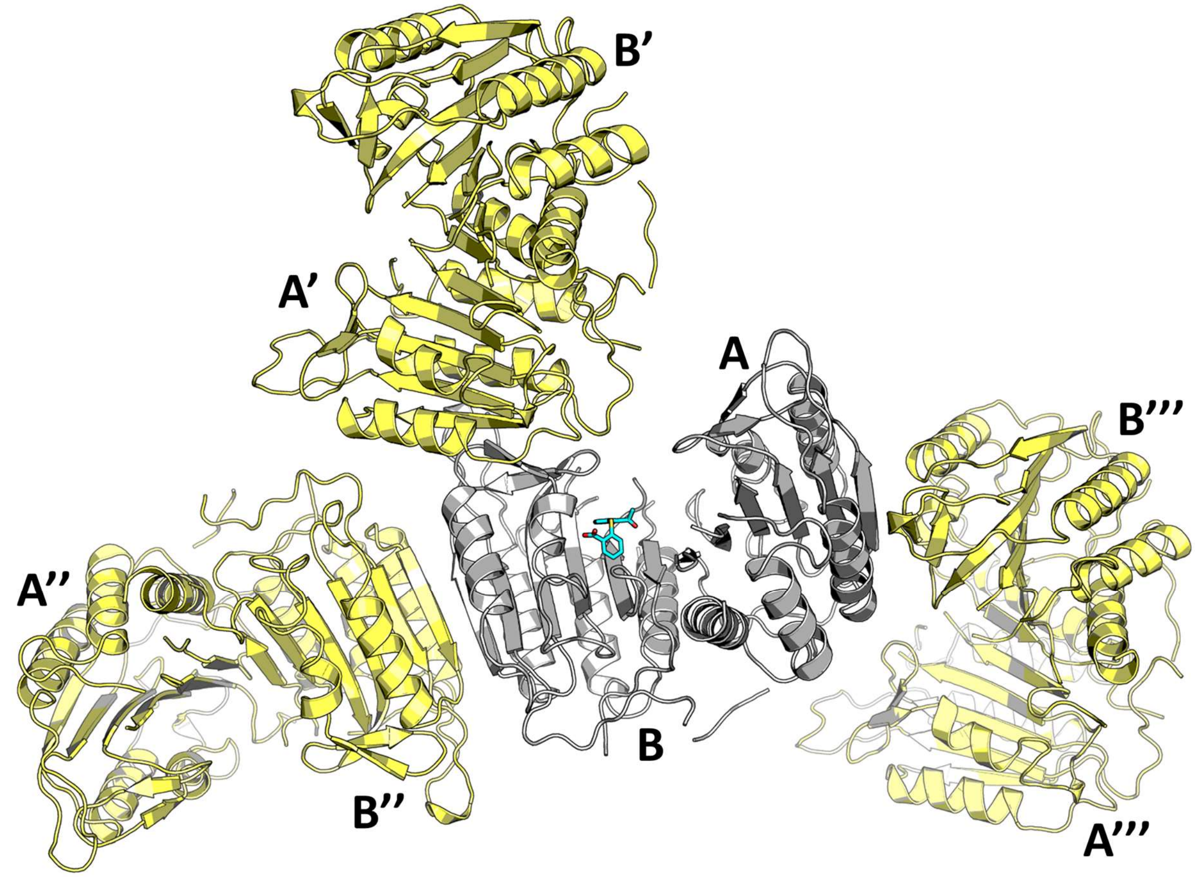

Figure S5: Comparison of B-factors between chains A and B of C7 soaked with **1** bound to the more ordered monomer B. Substrate binding loops L1, L2, L3, and L4 are highlighted by black bars above residue numbers. (PDB ID: 5V6U)

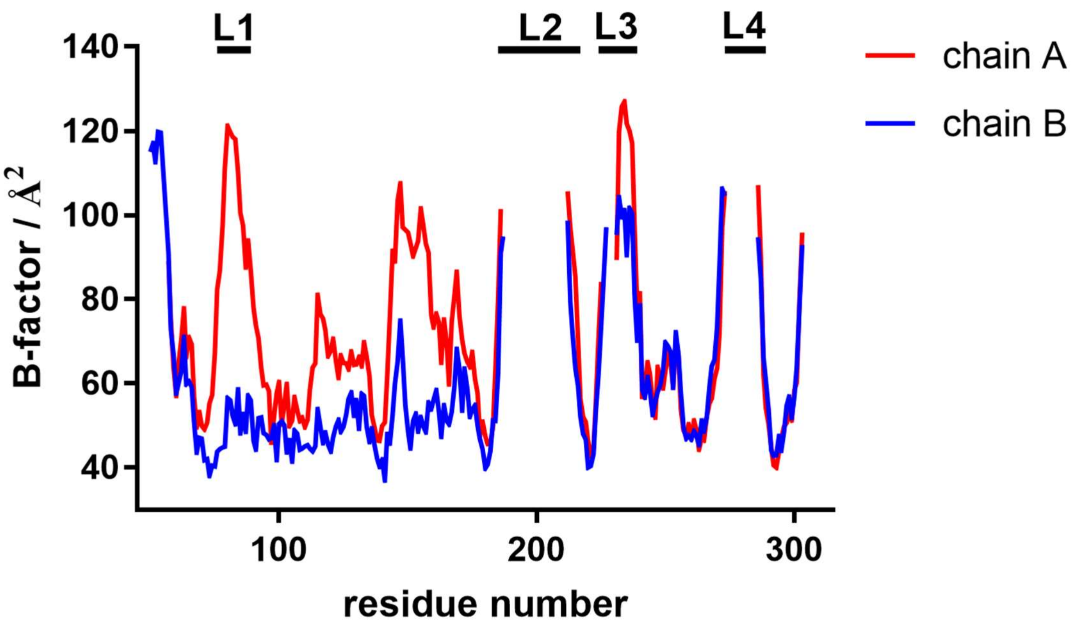

Figure S6: Comparison of B-factors between chains A and B of mature C7. Substrate binding loops L1, L2, L3 and L4 are highlighted by black bars above residue numbers. (PDB ID: 4FDL)

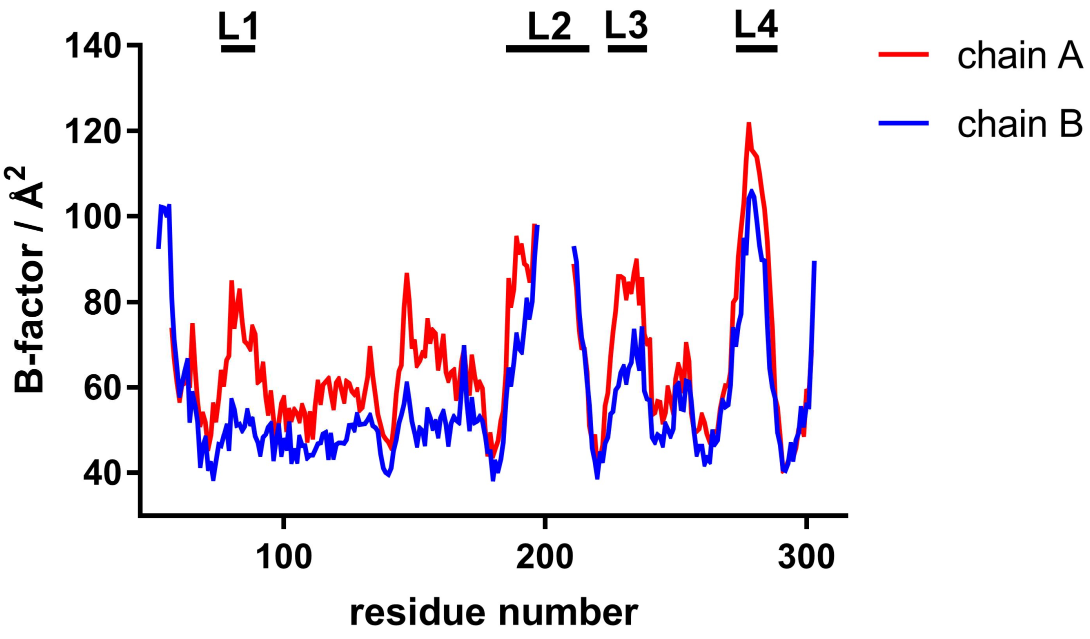

Figure S7: k_{cat}/K_m vs pH profile for C7 and P7-D_2A

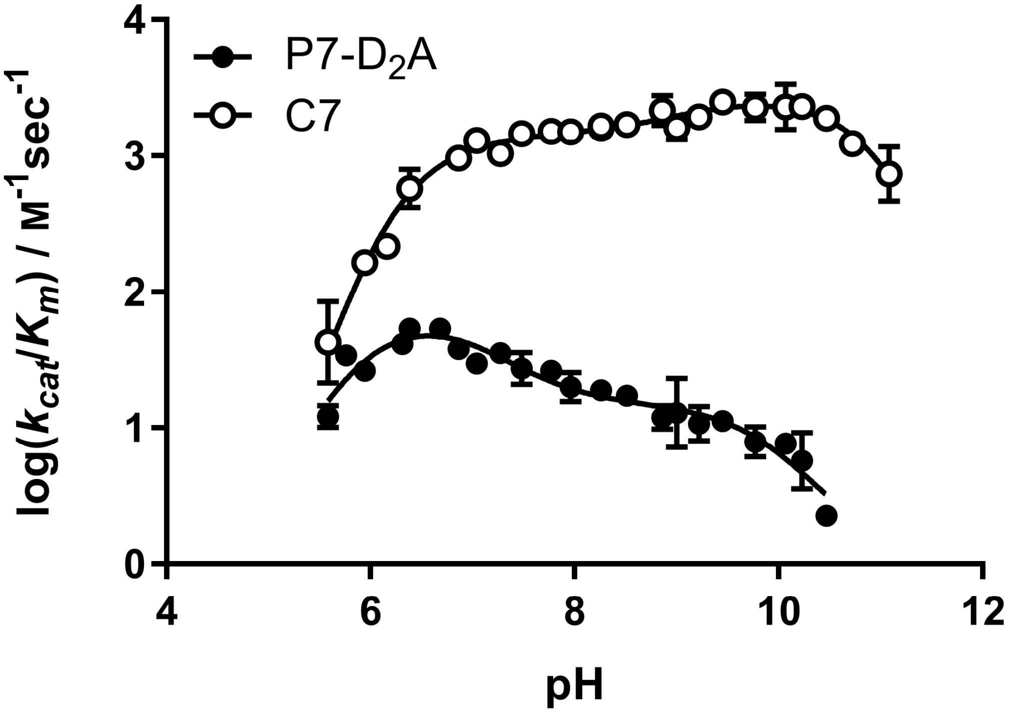

Figure S8: k_{cat} vs pH profile for C7 and P7-D_2A

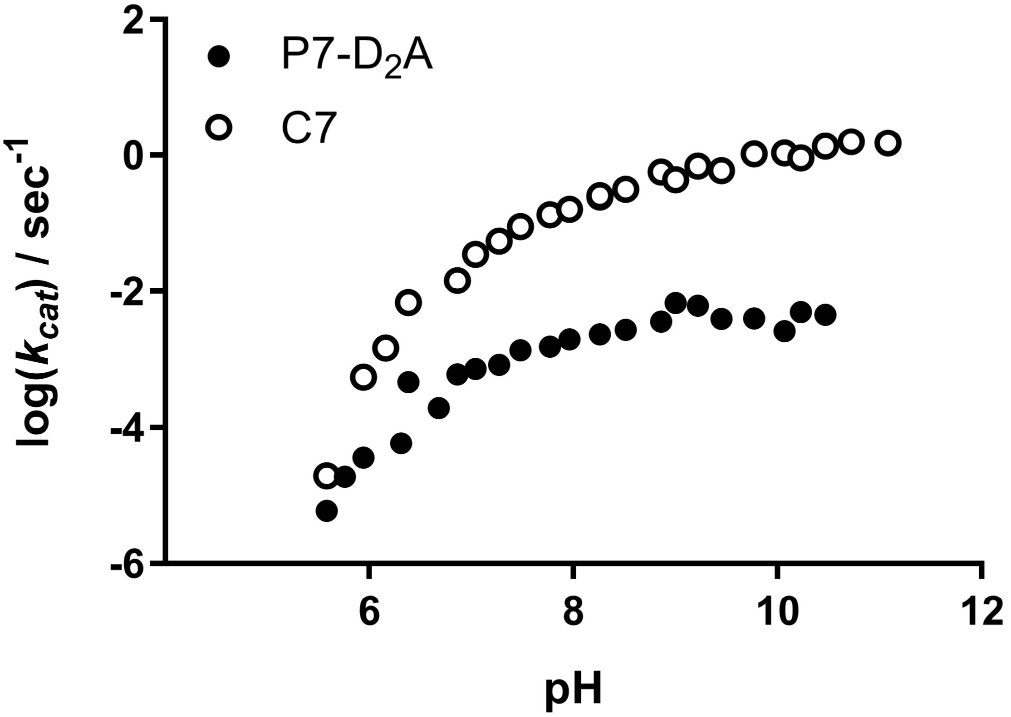

Figure S9: K_m vs pH profile for C7 and P7-D$_2$A

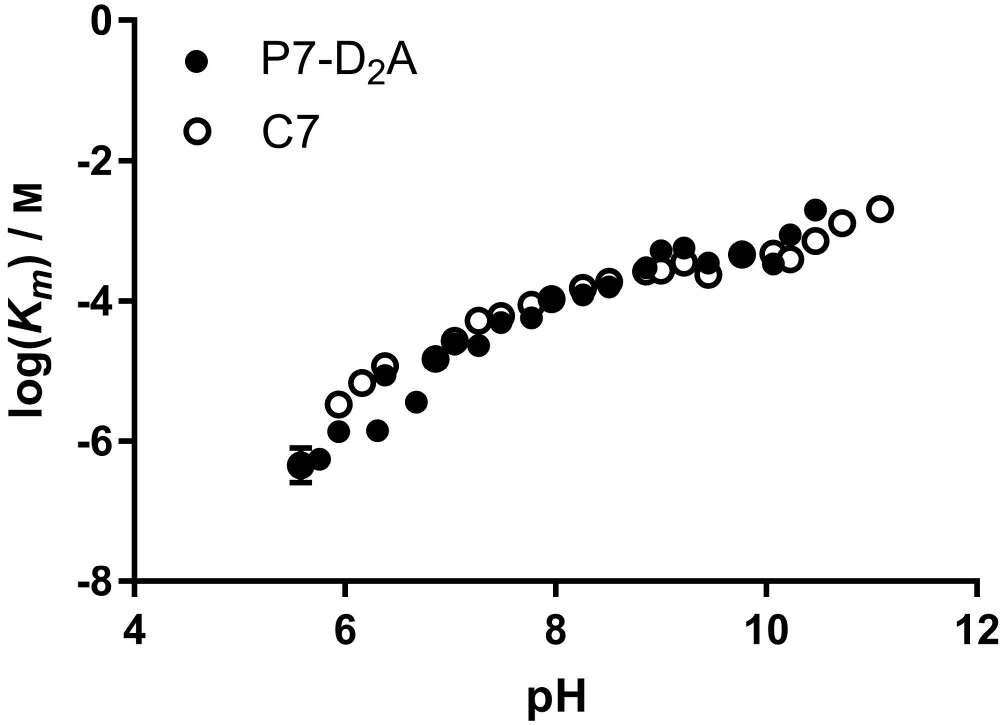

Table S1. OneStep data for **1** and **2** was analysed by fitting with a simple 1:1 binding model and fit in Qdat to determine the micro- and macroscopic binding rate constants

Compound	k_a [M $^{-1}$sec^{-1}]	k_d [sec^{-1}]	K_D [mM]	RU$_{max}$	Res. S.D.
1	0.083 ± 2x10^{-3}	7.0 ± 2x10^{-4}	8.0 ± 3.0	160	2.438
2	0.640 ± 2x10^{-3}	2.61 ± 3x10^{-5}	4.09 ± 0.03	160	13.067

[a] Res. S.D. = Residual Standard Deviation

Table S2. Data collection and refinement statistics

	**Caspase7_compound1**
**PDB ID**	5V6U
	**Data collection**
Space group	P 3_2 2 1
Unit cell parameters (a, b, c) [Å]<br>(α, β, γ) [º]	88.39 88.39 185.36<br>90.00 90.00 120.00
R_{merge} / R_{rim} / R_{pim}	0.105 / 0.116 / 0.049
$\langle I/\sigma(I) \rangle$	19.3 (2.2)
Completeness (%)	99.8 (48.08 – 2.80)
Multiplicity	10.4
Reflections (total / unique)	222,197 / 21,407
Solvent content	59.6
Mosaicity (º)	0.09
Wilson B factor [Å^2]	63.4
Monomers per ASU	2
Wavelength [Å]	1.00
X-ray source	ALS beamline 4.2.2
	**Refinement**
Resolution [Å]	48.08 - 2.80
R_{work}/R_{free} [%]	19.1 / 24.5
**Number atoms**	
Protein	3222 (418 residues)
Ligand	19
Water	76
**R.m.s. deviations**	
Bond lengths [Å]	0.014
Bond angles [º]	1.498
**Average B-factor**	
Protein [Å^2] (chain A / B)	69.85 / 58.41
Ligand [Å^2]	70.20
Water [Å^2]	61.11
**Ramachandran Plot**	
Favored / Allowed / Disallowed [%]	96.0 / 4.0 / 0.0

Table S3. Solvent isotope effects for C7 and P7-D_2A in H_2O and D_2O, using the substrate Ac-DEVD-AFC.

Enzyme	$^{D}k_{cat}$	$^{D}(k_{cat}/K_m)$
C7	4.11 ± 0.01	0.92 ± 0.04
P7-D_2A	2.93 ± 0.01	0.43 ± 0.04

Table S4. Michaelis menten parameters for C7 and P7-D_2A in H_2O and D_2O, using the substrate Ac-DEVD-AFC.

Solvent	Enzyme	k_{cat} [sec^{-1}]	K_m [μM]	k_{cat}/K_m [$M^{-1}sec^{-1}$]
H_2O	C7	0.0397 ± $2x10^{-5}$	45.45 ± 0.78	872.6 ± 14.99
D_2O	C7	0.0096 ± $4x10^{-6}$	10.15 ± 0.40	950.62 ± 37.85
H_2O	P7-D_2A	0.0032 ± $1x10^{-5}$	81.25 ± 4.79	38.80 ± 2.29
D_2O	P7-D_2A	0.0011 ± $2x10^{-6}$	12.09 ± 0.76	89.00 ± 5.62

Characterization of compounds

Compounds **1**, **2**, and **6** were purchased from Maybridge. Compounds **3**, **4**, and **5** were purchased from Enamine, Cayman Chemical and Vitas-M Laboratory, respectively. Compounds were used without further purification. Identity and purity of these compounds was confirmed by ^{1}H NMR in ^{2}H-DMSO and LC-MS. Compounds **2** and **6** undergo decarboxylation during ionization for LC-MS, in both positive and negative ion mode. ^{1}H NMR data show the carboxyl group is still intact, evidenced by the 12-14 ppm peak in all spectra, the aliphatic carboxyl group in **2** and **6** seem to be less stable to ionization compared to the aromatic carboxyls.

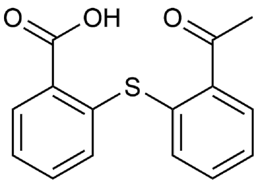	compound 1 (JFD00837)<br>2-((2-acetylphenyl)thio)benzoic acid<br>Chemical Formula: $C_{15}H_{12}O_3S$<br>Exact Mass: 272.05<br>Molecular Weight: 272.32<br>m/z: 272.05 (100.0%), 273.05 (16.2%), 274.05 (4.5%), 274.06 (1.2%)
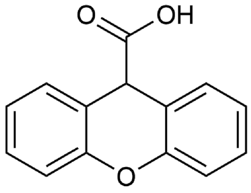	compound 2 (RH00001)<br>9*H*-xanthene-9-carboxylic acid<br>Chemical Formula: $C_{14}H_{10}O_3$<br>Exact Mass: 226.06<br>Molecular Weight: 226.23<br>m/z: 226.06 (100.0%), 227.07 (15.1%), 228.07 (1.1%)
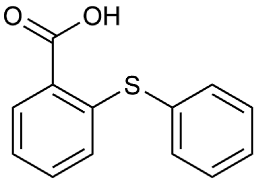	compound 3 (EN300-91827)<br>2-(phenylthio)benzoic acid<br>Chemical Formula: $C_{13}H_{10}O_2S$<br>Exact Mass: 230.04<br>Molecular Weight: 230.28<br>m/z: 230.04 (100.0%), 231.04 (14.1%), 232.04 (4.5%)
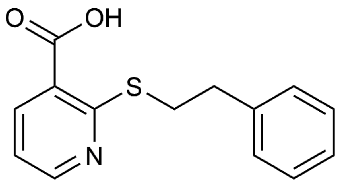	compound 4 (ML-099)<br>2-(phenethylthio)nicotinic acid<br>Chemical Formula: $C_{14}H_{13}NO_2S$<br>Exact Mass: 259.07<br>Molecular Weight: 259.32<br>m/z: 259.07 (100.0%), 260.07 (15.1%), 261.06 (4.5%), 261.07 (1.1%)
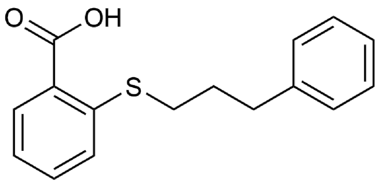	compound 5 (STK288473)<br>2-((3-phenylpropyl)thio)benzoic acid<br>Chemical Formula: $C_{16}H_{16}O_2S$<br>Exact Mass: 272.09<br>Molecular Weight: 272.36<br>m/z: 272.09 (100.0%), 273.09 (17.3%), 274.08 (4.5%), 274.09 (1.4%)
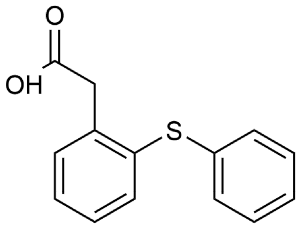	compound 6 (BTB15078)<br>2-(2-(phenylthio)phenyl)acetic acid<br>Chemical Formula: $C_{14}H_{12}O_2S$<br>Exact Mass: 244.06<br>Molecular Weight: 244.31<br>m/z: 244.06 (100.0%), 245.06 (15.1%), 246.05 (4.5%), 246.06 (1.1%)

Physicochemical properties of compounds

Depictions and physicochemical property calculations for the compounds were done with ChemDraw (Perkin-Elmer).

Compound	MW [Da]	cLogP	tPSA [Å^2]	LogS	LogD	pK_a
1	272.05	3.10	54.37	-4.20	0.08	3.32
2	226.06	3.10	46.53	-3.10	-0.31	3.33
3	230.04	3.60	37.30	-4.00	0.52	3.57
4	259.07	3.34	49.66	-3.50	0.42	3.26
5	272.09	4.28	37.30	-4.56	1.36	3.54
6	244.31	3.75	37.30	-3.87	1.04	4.10

Compound **1** ^{1}H-NMR

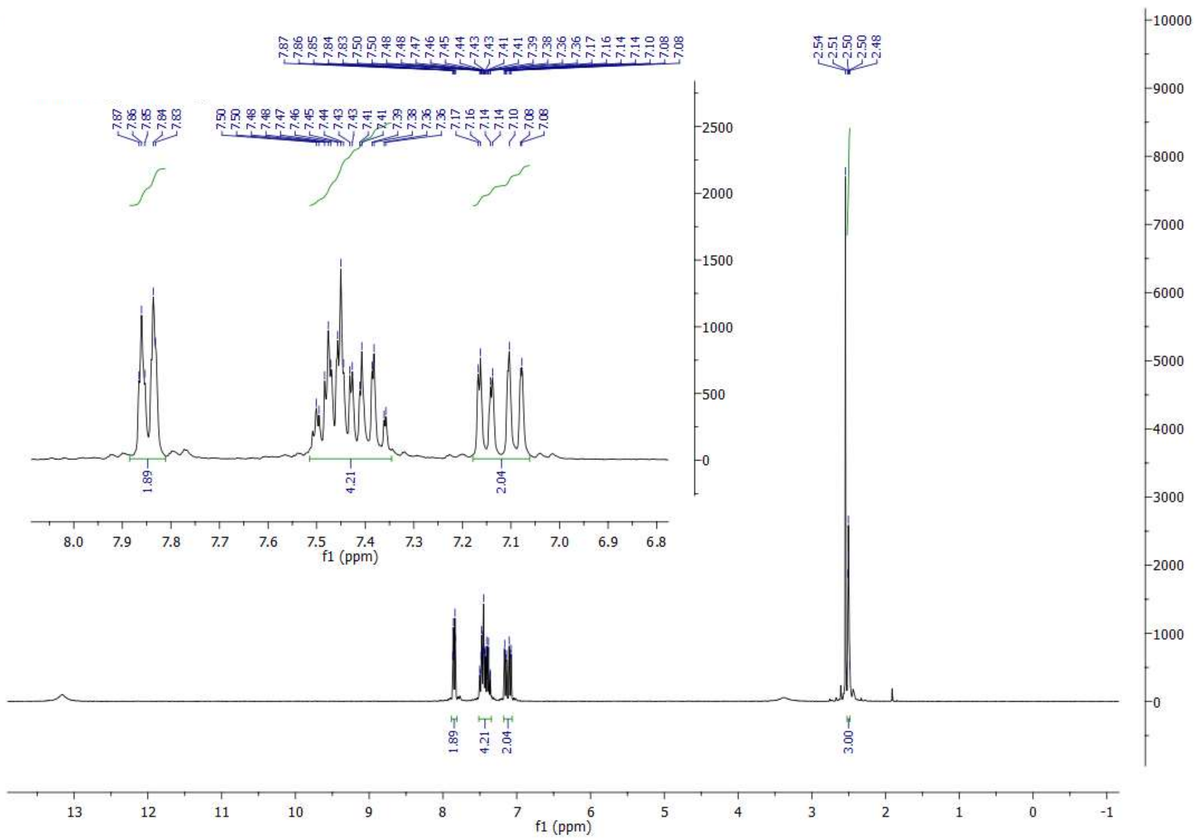

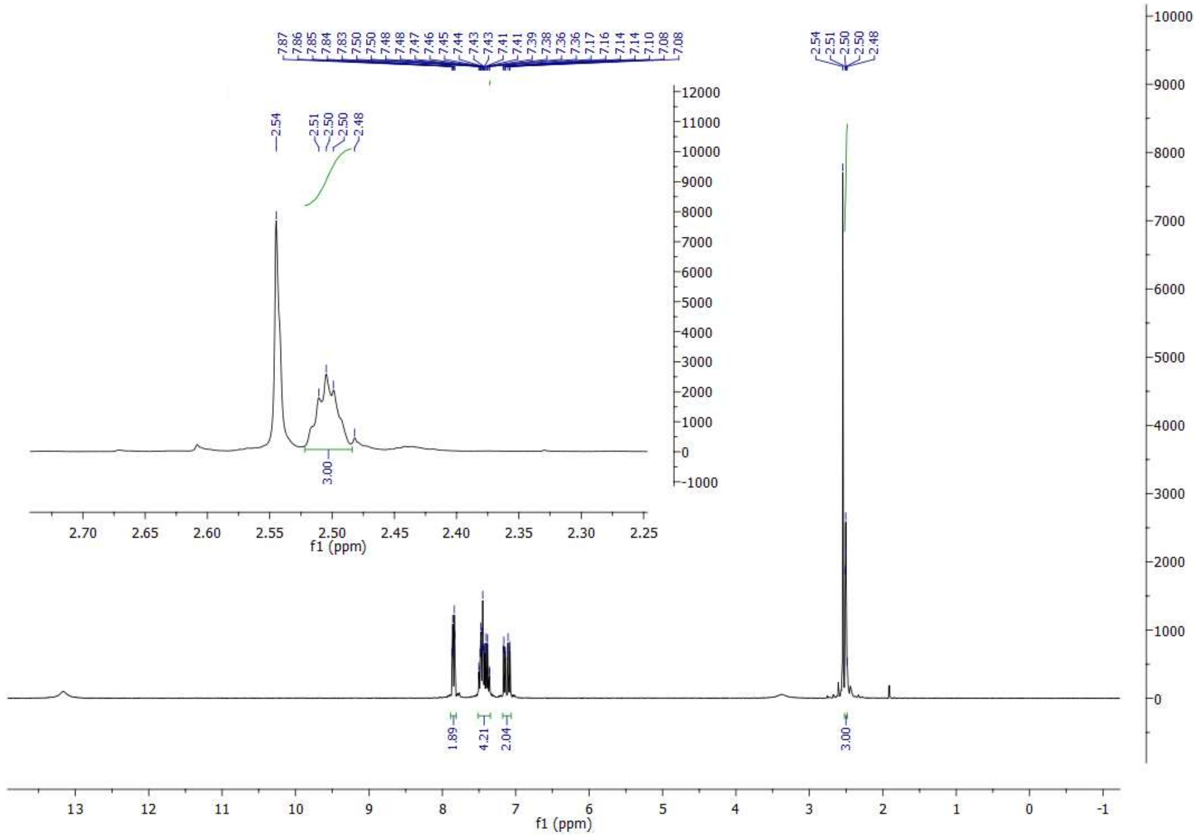

Compound **1** LC-MS

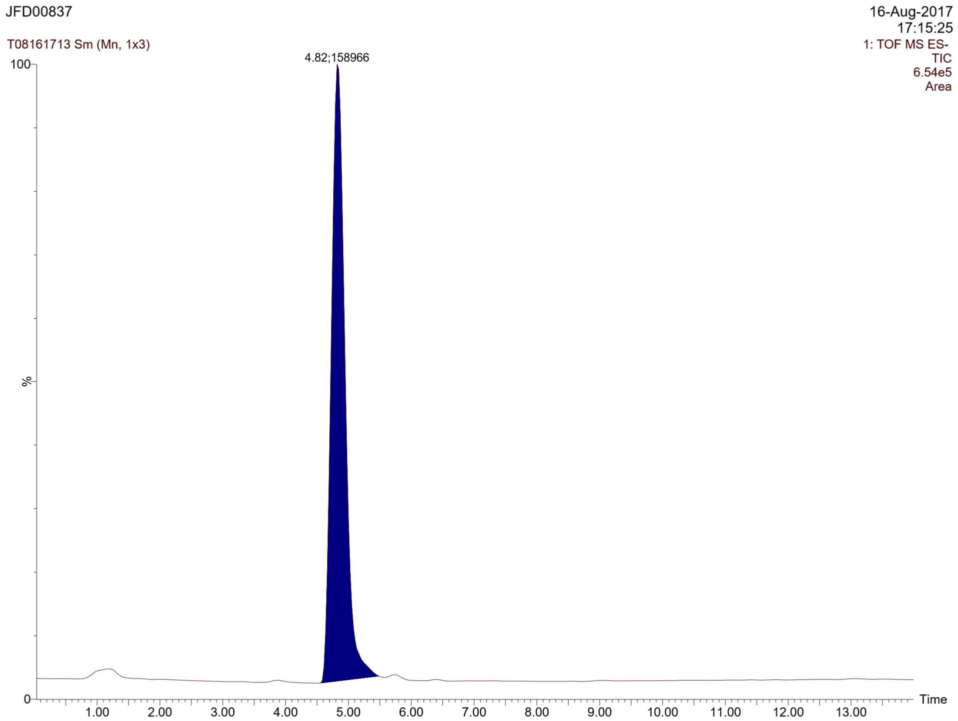

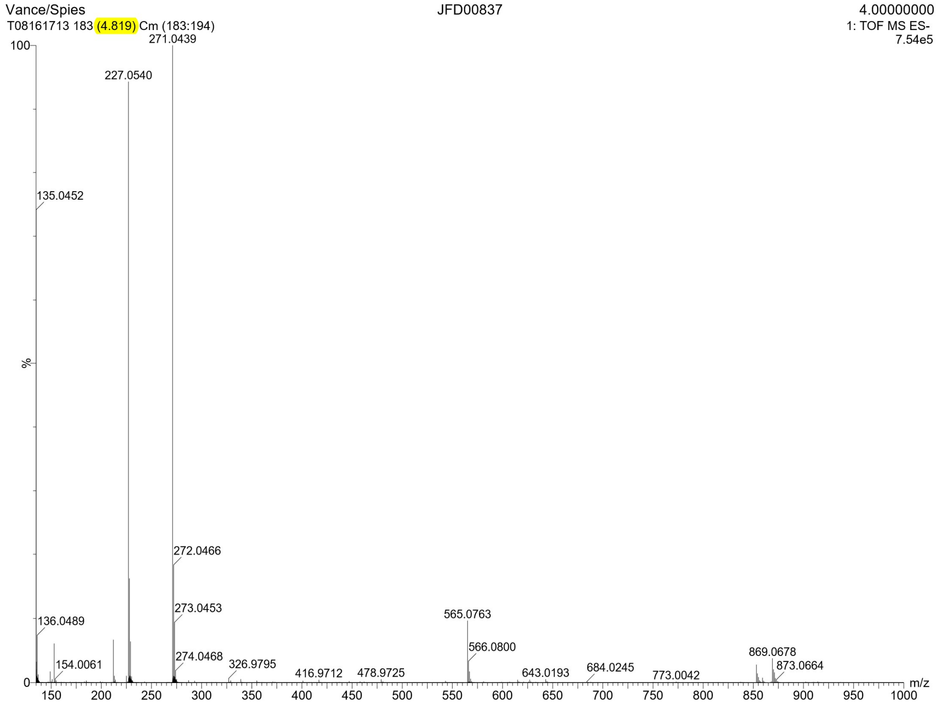

Compound **2** ^{1}H-NMR

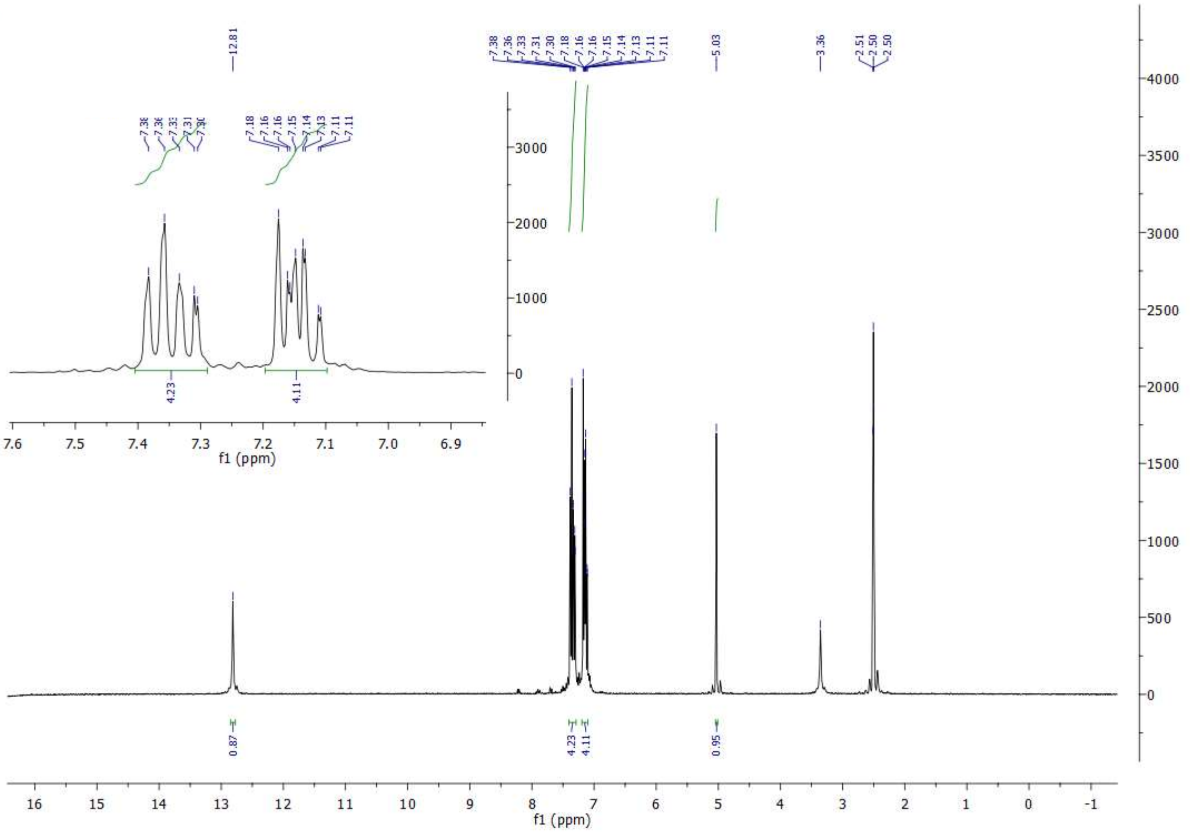

Compound **2** LC-MS

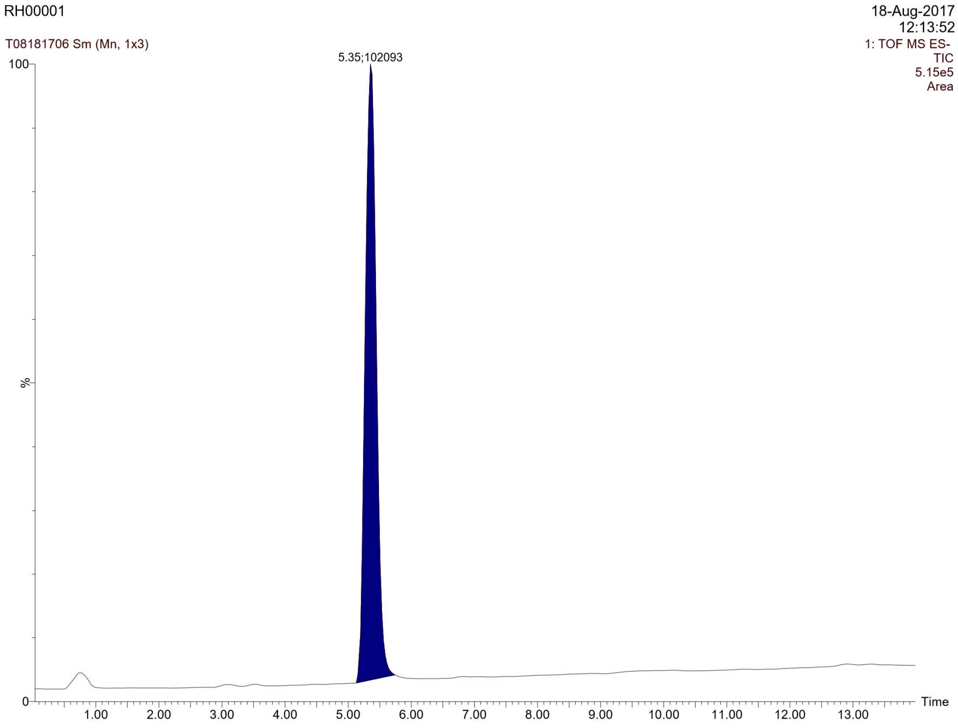

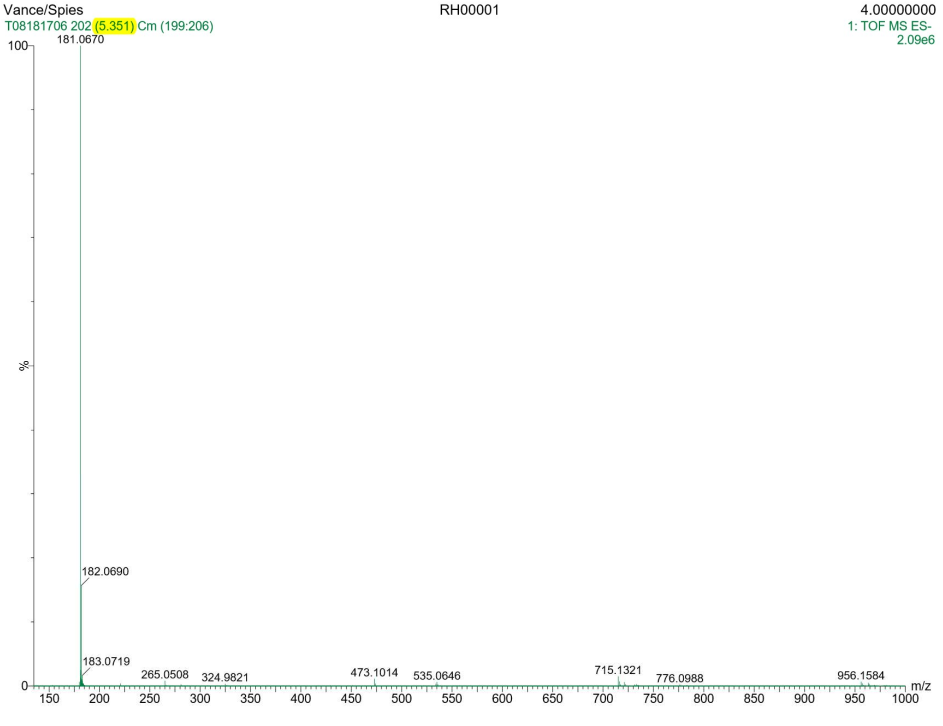

Compound **3** ^{1}H-NMR

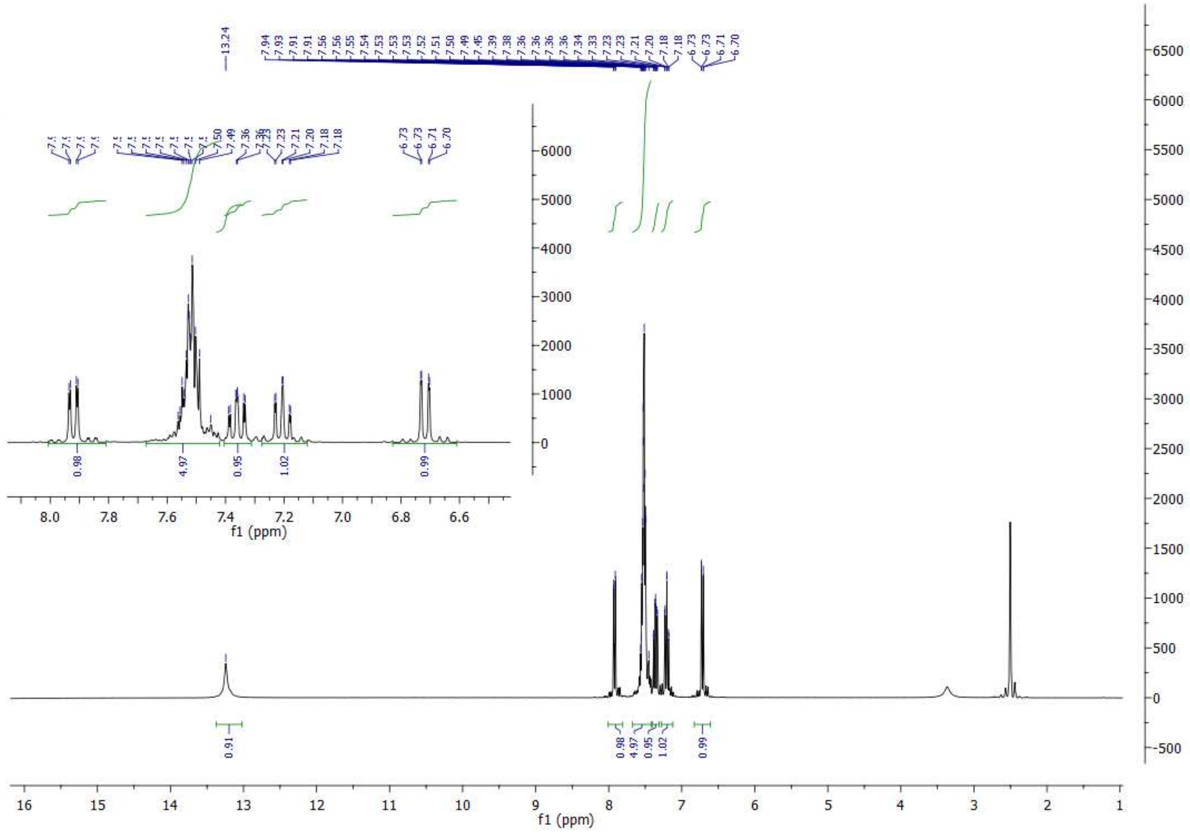

Compound **3** LC-MS

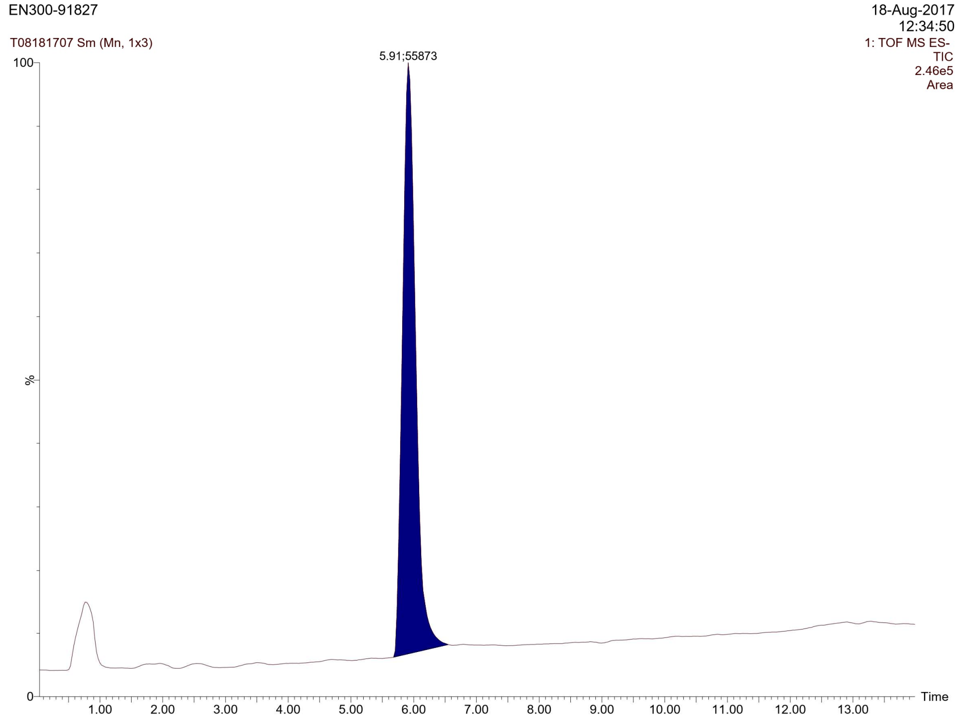

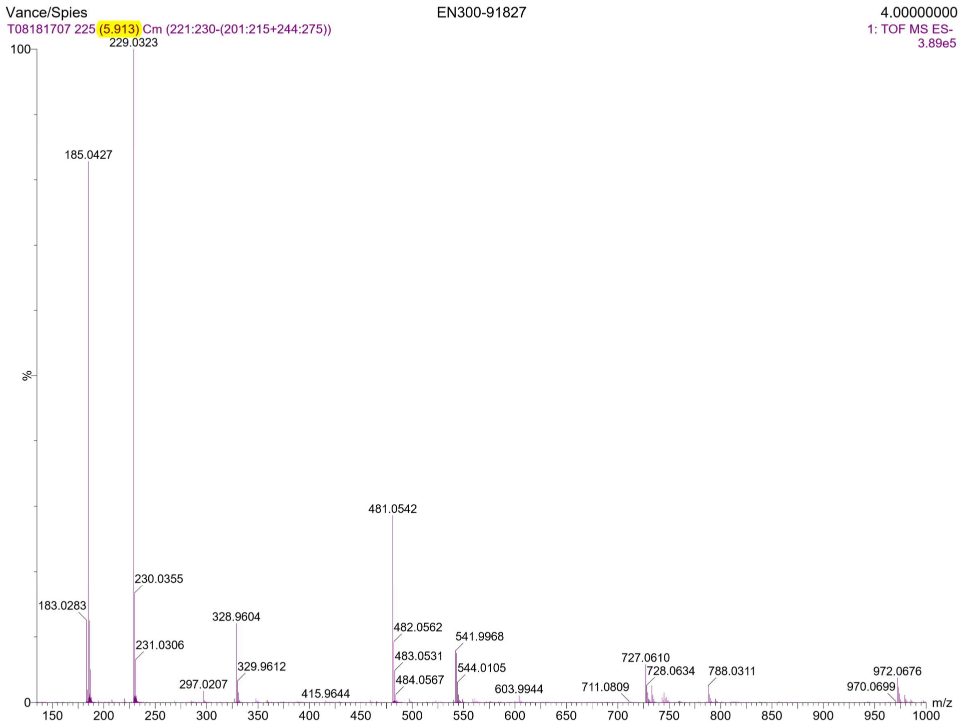

Compound **4** ^{1}H-NMR

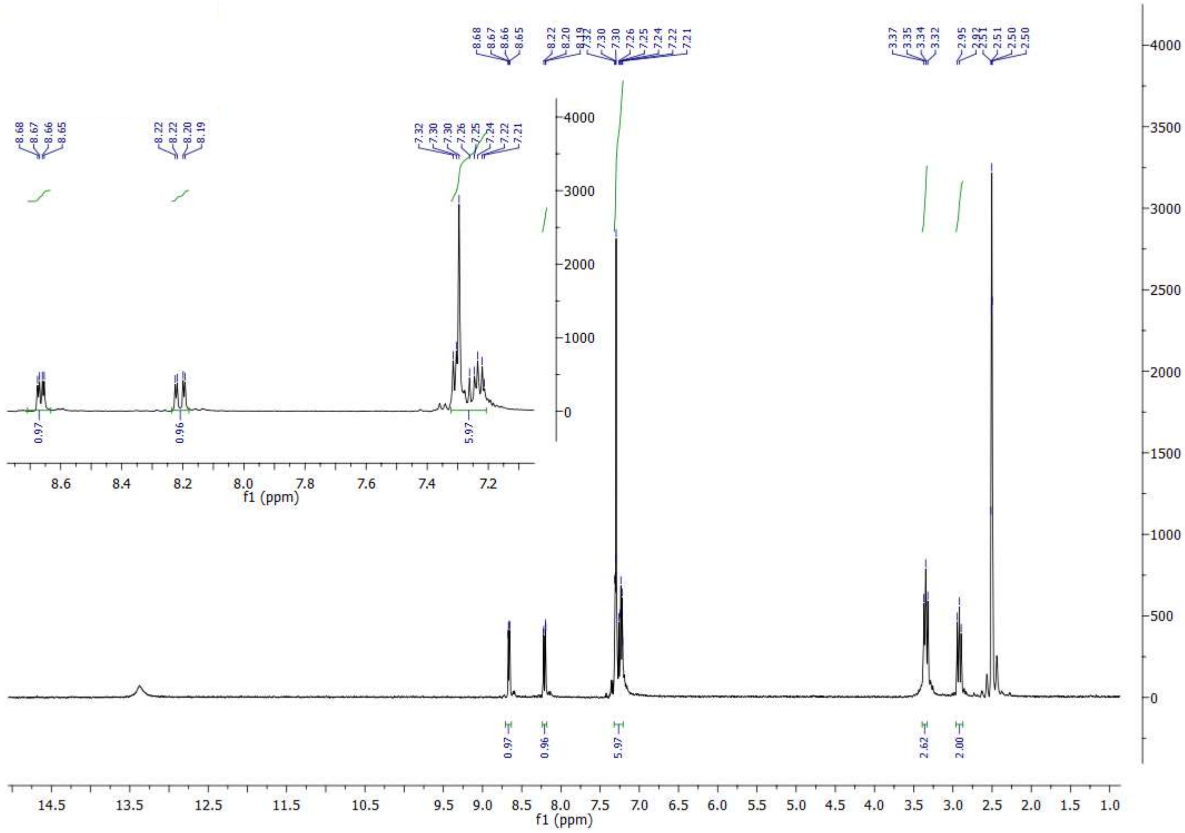

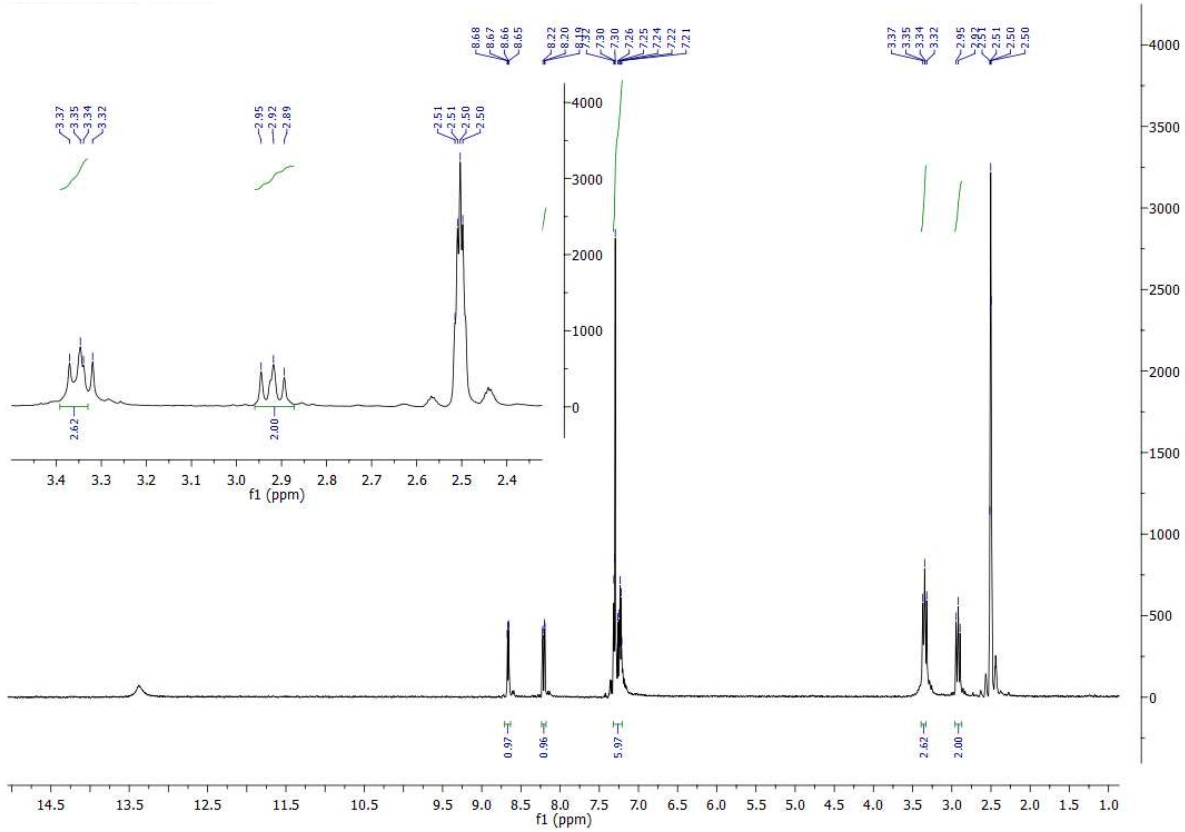

Compound **4** LC-MS

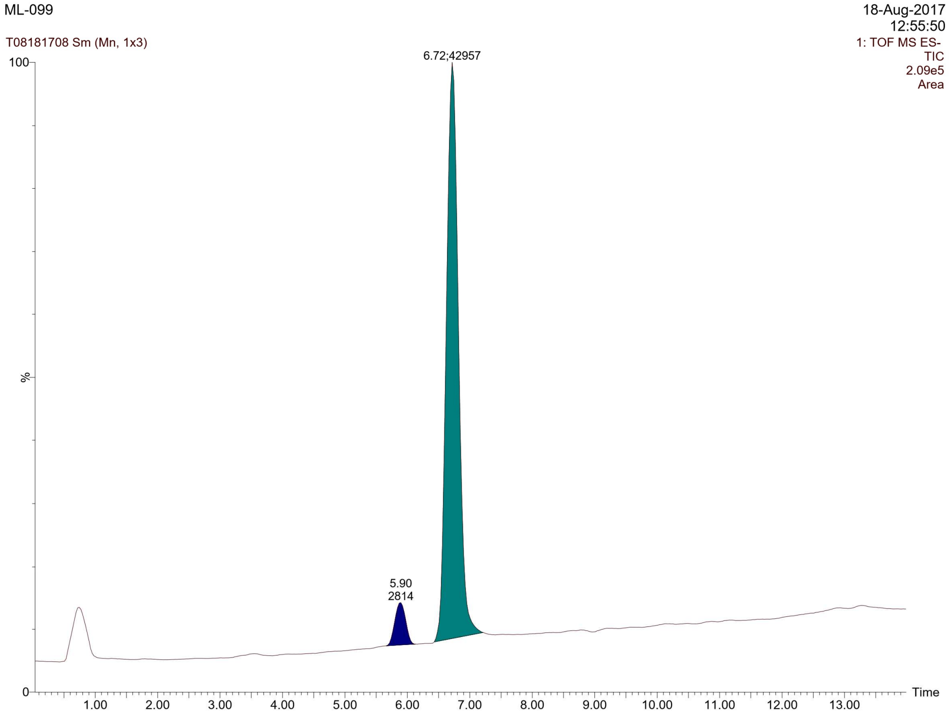

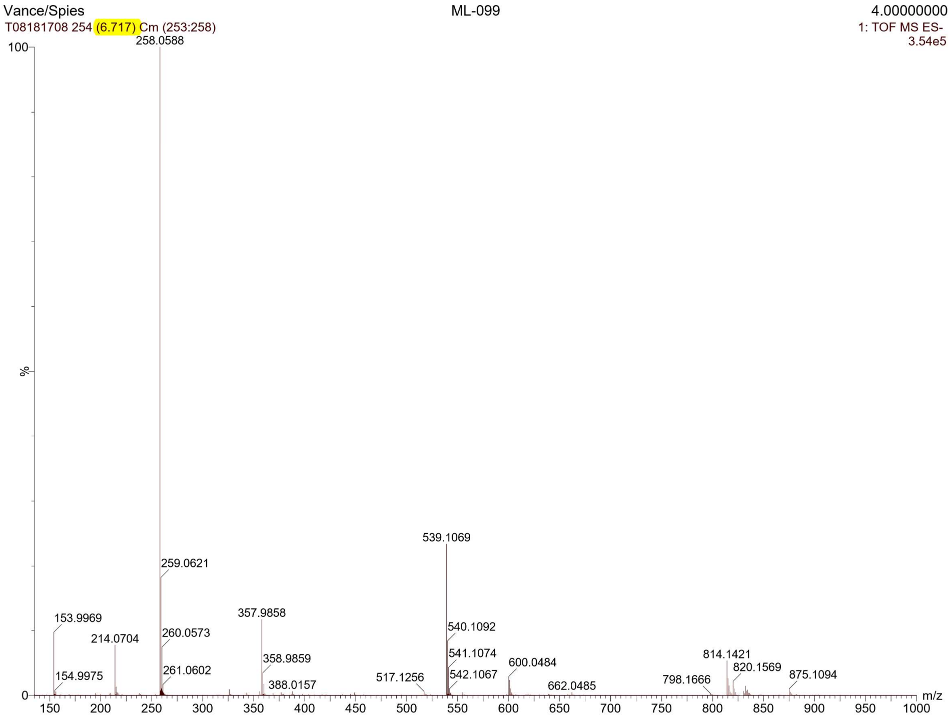

Compound **5** ^{1}H-NMR

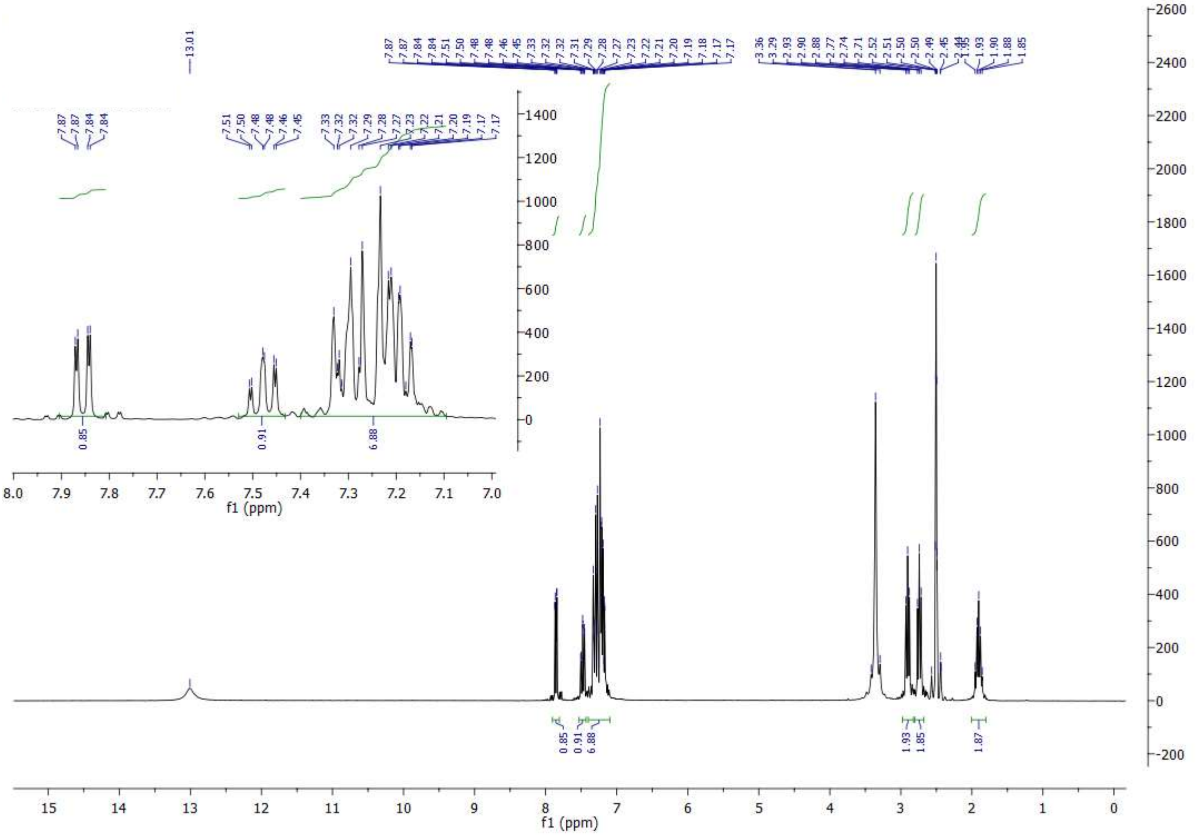

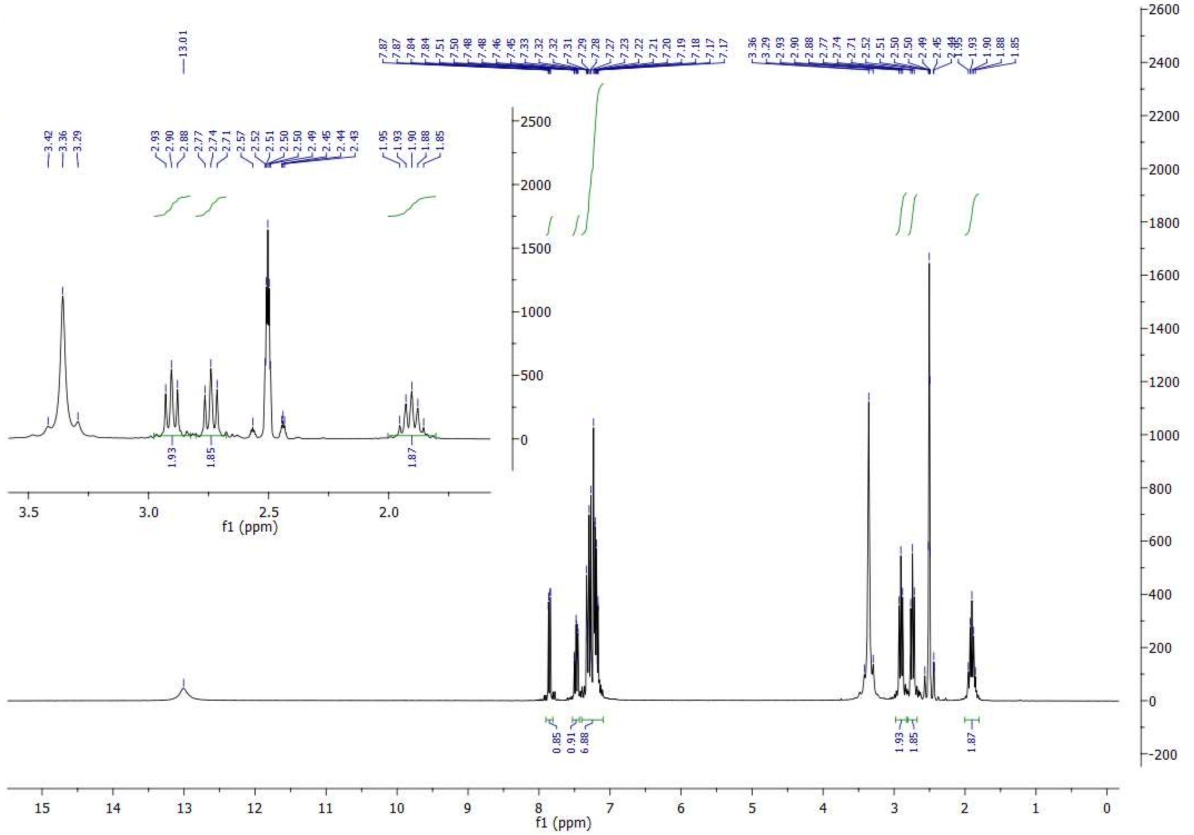

Compound **5** LC-MS

STK288473

18-Aug-2017
18:50:42

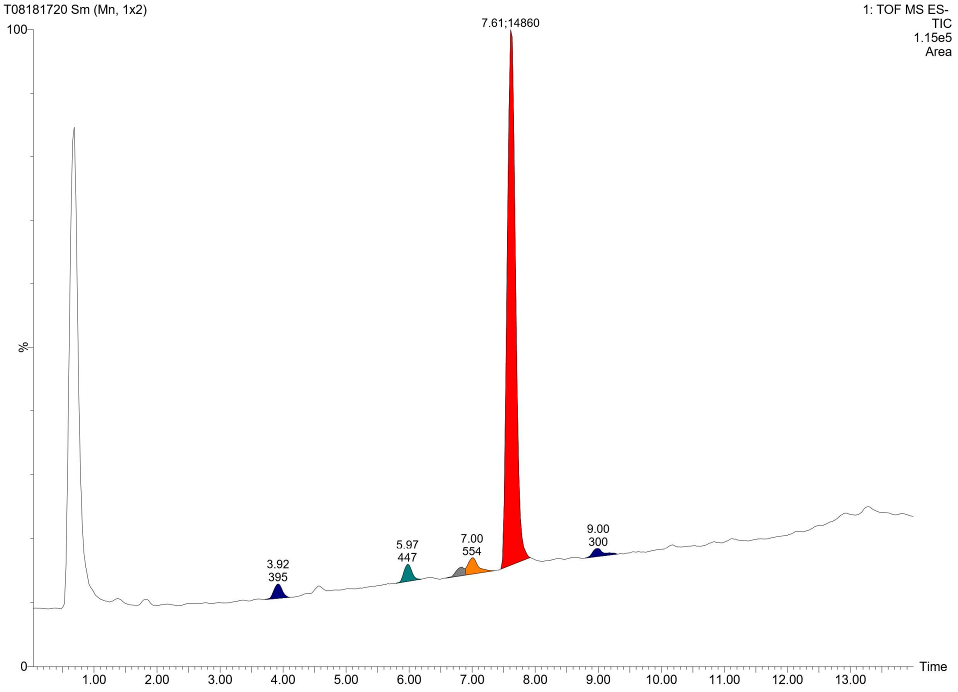

Vance/Spies

STK288473

4.00000000

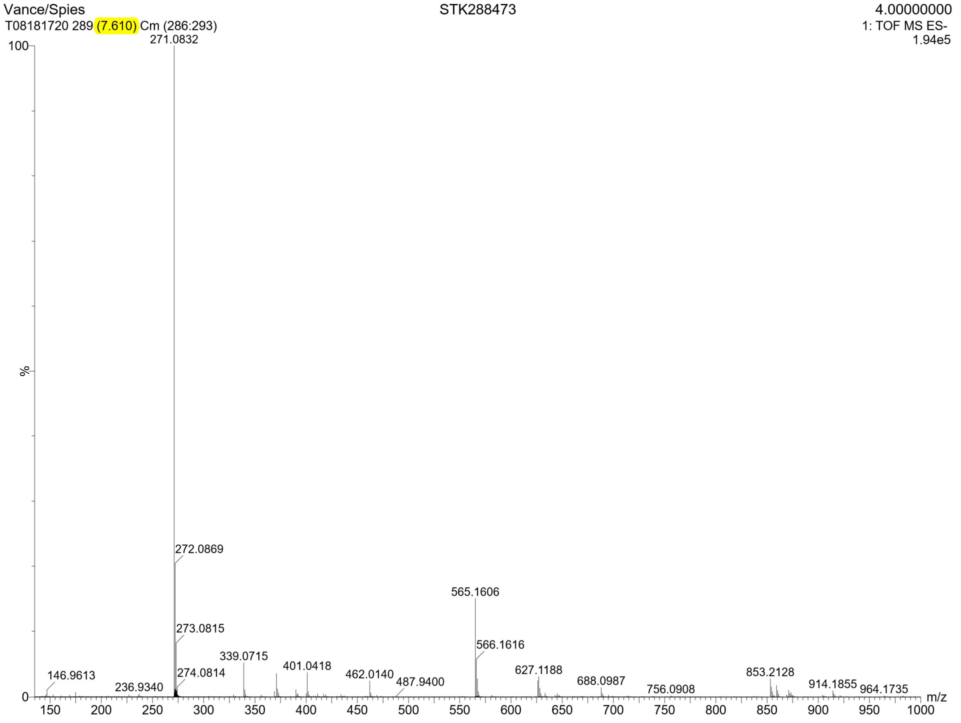

Compound **6** ^{1}H-NMR

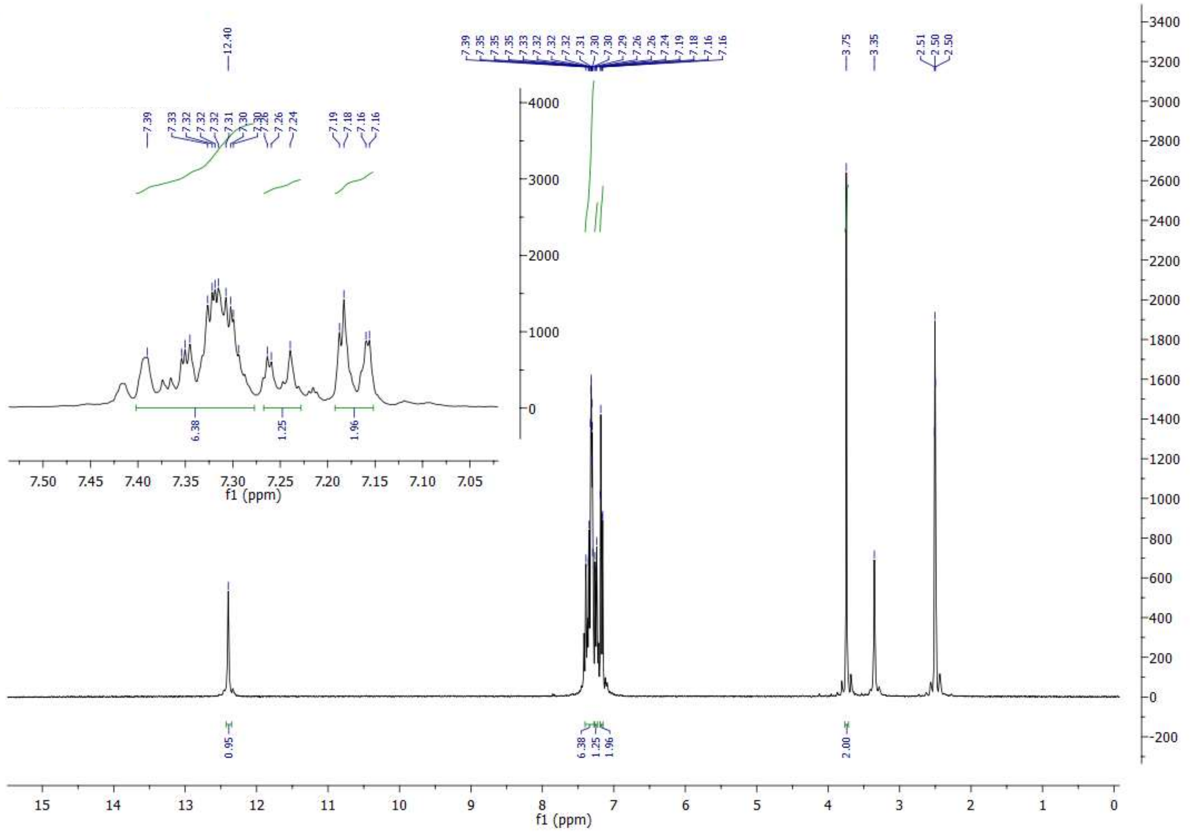

Compound **6** LC-MS

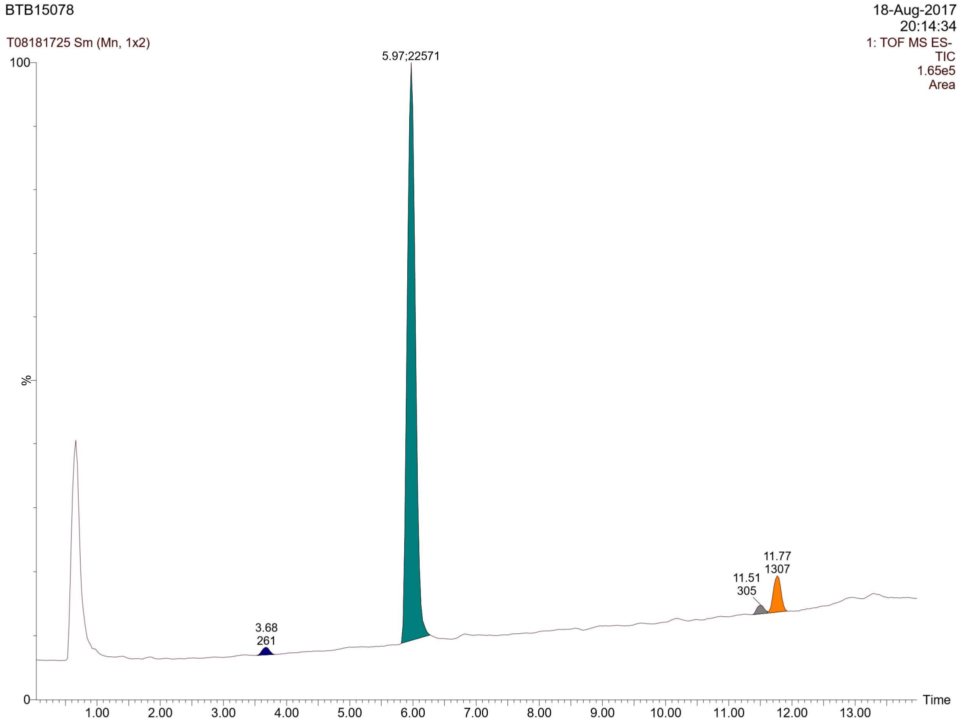

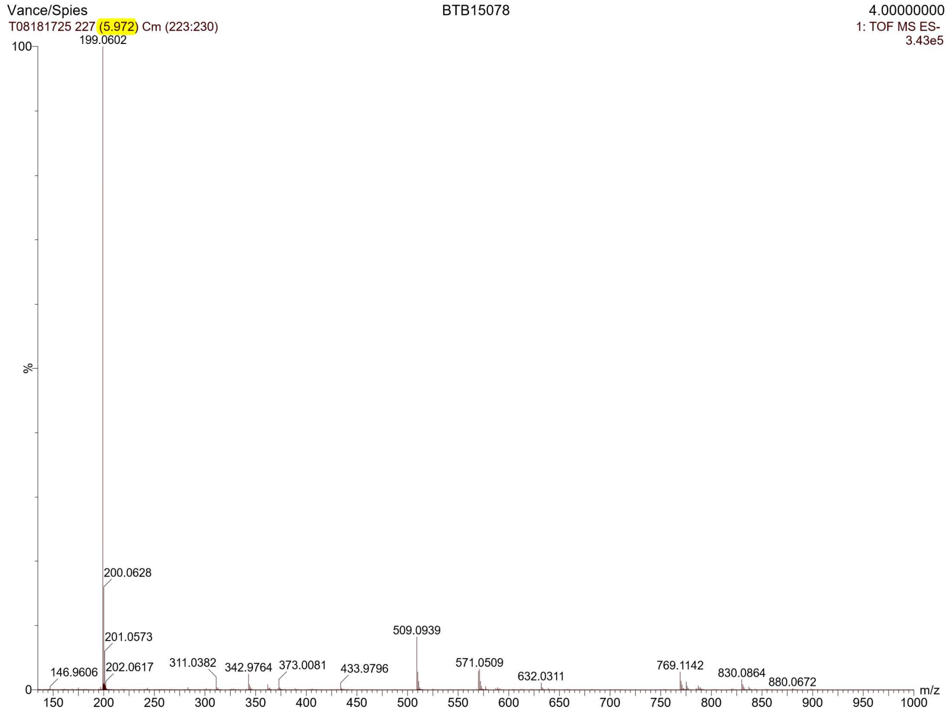

Supplement: Supplementary file 1 — Supplementary [file ANIE-56-14443-s001.pdf]
